# S-adenosyl-L-methionine is the unexpected methyl donor for the methylation of mercury by the membrane-associated HgcAB complex

**DOI:** 10.1073/pnas.2408086121

**Published:** 2024-11-15

**Authors:** Kaiyuan Zheng, Katherine W. Rush, Swapneeta S. Date, Alexander Johs, Jerry M. Parks, Angela S. Fleischhacker, Macon J. Abernathy, Ritimukta Sarangi, Stephen W. Ragsdale

**Affiliations:** ^a^Department of Biological Chemistry, University of Michigan Medical School, Ann Arbor, MI 48109-0606; ^b^Environmental Sciences Division, Oak Ridge National Laboratory, Oak Ridge, TN 37831-6038; ^c^Biosciences Division, Oak Ridge National Laboratory, Oak Ridge, TN 37831-6309; ^d^Department of Structural Molecular Biology, Stanford Synchrotron Radiation Lightsource, SLAC National Accelerator Laboratory, Menlo Park, CA 94025

**Keywords:** methyl mercury, S-adenosyl-L-methionine, cobalamin, iron-sulfur, methyltransferase

## Abstract

Mercury (Hg) is a heavy metal that exhibits high biological toxicity. Monomethylmercury and dimethylmercury are neurotoxins and a significant environmental concern as they bioaccumulate and biomagnify within the aquatic food web. Microbial Hg methylation involves two proteins, HgcA and HgcB. Here, we show that HgcA and HgcB can be heterologously coexpressed, and the HgcAB complex can be purified. We demonstrated that HgcA is a membrane-associated cobalamin-dependent methyltransferase and HgcB is a ferredoxin-like protein containing two [4Fe-4S] clusters. Further, spectroscopic and kinetic results demonstrate that S-adenosyl-L-methionine (SAM) donates the methyl group to Hg in a two-step reaction involving a methylcob(III)alamin intermediate including Co-thiolate ligation from a conserved Cys residue. Our findings uncover a biological role for SAM in microbial Hg methylation.

## Significance

The microbial conversion of inorganic mercury to highly neurotoxic methylmercury is a global environmental concern and a threat to human health. Most mercury methylation research is focused on genomic and microbial aspects, and biochemical studies have been challenging because of the difficulties in expressing and purifying the proteins involved in mercury methylation (HgcAB). The present study establishes the active expression, purification, and characterization of HgcAB as a B_12_/iron–sulfur enzyme that catalyzes S-adenosyl methionine (SAM)-dependent Hg methylation through a methyl-Co-thiolate intermediate. This work identifies a metabolic role for SAM in heavy metal–associated biological processes.

Mercury (Hg) is a pervasive global pollutant and health threat that predominantly occurs in the environment as elemental Hg^0^, Hg^++^, and its highly toxic forms, methylmercury (CH_3_Hg^+^, MeHg) and dimethylmercury [Hg(CH_3_)_2_, Me_2_Hg] (1). Hg is released into ecosystems from natural geothermal activity as well as from anthropogenic sources, including mining operations, coal combustion, and chemical industries (2). Microorganisms convert Hg^++^ into MeHg, which bioaccumulates and biomagnifies up trophic levels throughout the food chain (3–5) and is responsible for Minamata disease (6). Understanding the fundamental mechanism of MeHg formation will promote the development of effective strategies to combat exposure to this neurotoxin and may provide insights into how other heavy metal(loid) (i.e., As, Sn) homeostasis pathways produce harmful methylated metal(loid) products.

Work in the early 1990s indicated that MeHg formation is a cobalamin-dependent enzymatic process (7). More recently, the two genes, *hgcA* and *hgcB*, involved in mercury methylation were identified (3) and subsequent phylogenic analyses identified mercury-methylating microorganisms within many phyla, including sulfate- and iron-reducing bacteria, Deltaproteobacteria, Firmicutes, Geobacterales, Nitrospirae, Spirochaetes, Actinobacteria, and Euryarchaeota (8–10), but with no obvious metabolic trends (11–13). Bioinformatic analyses and structural modeling (14) indicate that HgcA consists of an N-terminal cobalamin binding domain and a C-terminal transmembrane domain consisting of five transmembrane α-helices (3), while HgcB contains two CxxCxxCxxxCP motifs proposed to bind [4Fe-4S] clusters (14–17). Mutagenesis studies identified a conserved cysteine residue (C93) in HgcA (18) and three cysteine residues on HgcB (C73, C94, and C95) that appear to be crucial for mercury methylation activity (3, 18). Moreover, recent sequence and codon analyses suggested that some homologs of *hgcA* and *hgcB* may be selenoproteins (19).

Methyltetrahydrofolate (Me-THF), a common methyl donor in cobalamin-dependent methyl transferases (20–22), was proposed to be the Hg methyl donor (7). DFT calculations suggested the reaction occurs through a methyl carbanion or radical intermediate (23, 24). Nevertheless, it has been challenging to mechanistically characterize the reaction(s) catalyzed by HgcAB due to its very low expression levels (~0.0004% of total protein) (25, 26), membrane association, oxygen sensitivity, and the need to incorporate cobalamin and Fe-S clusters for activity. Here, we report an expression and purification protocol for the HgcAB complex that improves yields and activity, allowing us to characterize the HgcAB complex, and demonstrate that SAM is the methyl donor.

## Results

### Purification of the HgcAB Complex.

While important strides have been made by microbiological, genetic, and bioinformatic studies, no successful purification protocol of the active HgcAB complex had been established. Prior mutagenesis studies indicated that the transmembrane domain of HgcA is required for Hg methylation activity (18). Furthermore, our previous efforts at independent heterologous expression of either HgcA or HgcB in *Escherichia coli* produced insoluble proteins lacking cobalamin, which indicated that we should coexpress HgcA and HgcB together via a N-terminal His-tagged full-length HgcA and a tagless HgcB. Thus, we cloned the His-tagged variant of the full-length *Pseudodesulfovibrio mercurii* sp. nov. strain ND132 (*P. mercurii*) (formerly *Desulfovibrio desulfuricans* ND132) (27), *hgcA* (His-*hgcA*) in tandem with *hgcB* into the pETDuet vector to generate pETDuet-His-*hgcA-hgc*B (28). To fully load HgcAB with its cobalamin center and two [4Fe-4S] clusters, we cotransformed the pETDuet-His-*hgcA*-*hgcB* vector with the pRKISC plasmid (29–31) for Fe-S cluster assembly (graciously provided by Yasuhiro Takahashi, Osaka University) and the pBtu plasmid for cobalamin uptake (32) vectors into *E. coli* C41(DE3), which often aids in expression of membrane-associated proteins, and BL21(DE3) strains (32). Both C41(DE3) and BL21(DE3) cells were cultivated under aerobic conditions to reach OD_600_ ~0.35 and induced by arabinose and then reach OD_600_ ~0.9 and induced by IPTG for the *btu-* and *isc*-operon-encoded proteins and for HgcAB overexpression under anaerobic conditions. While the C41 cell lysate did not exhibit mercury methylation activity, that from BL21 did catalyze MeHg formation (*SI Appendix*, Fig. S1).

Incorporation of an N-terminal His-tag on HgcA and inclusion of sodium *N*-dodecanoyl-*N*-methylglycinate (Sarkosyl) and Triton X-100 detergents in the buffers enabled one-step purification of the HgcAB complex using a Ni-NTA resin (33), as shown by native- and sodium dodecyl sulfate - polyacrylamide gel electrophoresis (SDS-PAGE) and tandem mass spectrometric (MS/MS) analyses (*SI Appendix*, Figs. S2 and S3*C*). After buffer exchange and dialysis to remove excess salt and detergent, HgcAB exhibited ultraviolet (UV)-visible spectra indicating high levels of cobalamin. Quantification of the cobalamin by conversion to dicyano-Co(III)balamin (34, 35) demonstrated 70% incorporation (*SI Appendix*, Fig. S3 *A* and *B*). Furthermore, electron paramagnetic resonance (EPR) spectral analysis of the dithionite-reduced protein indicated incorporation of two iron–sulfur clusters ([4Fe-4S]/Cbl ~1.72) (*SI Appendix*, Fig. S4), as predicted previously based on sequence analysis (3, 14).

We tried various detergents to stabilize and purify the active membrane-associated HgcAB complex and to perform the experiments described here. For example, SDS denatured HgcAB, while CHAPS and octylglucopyranoside caused HgcAB precipitation, preventing cobalamin incorporation. Using only Sarkosyl in purification allowed solubilization of HgcAB, enabling Ni-NTA affinity chromatography purification, but inhibited methylation of the purified protein complex. Triton X-100 alone also resolubilized and allowed purification of an HgcAB complex that is active in the first half-reaction, but the yield was extremely low compared to the Sarkosyl-Triton combination.

### Demonstration that SAM Is the Methyl Donor.

**Fig. 1.**
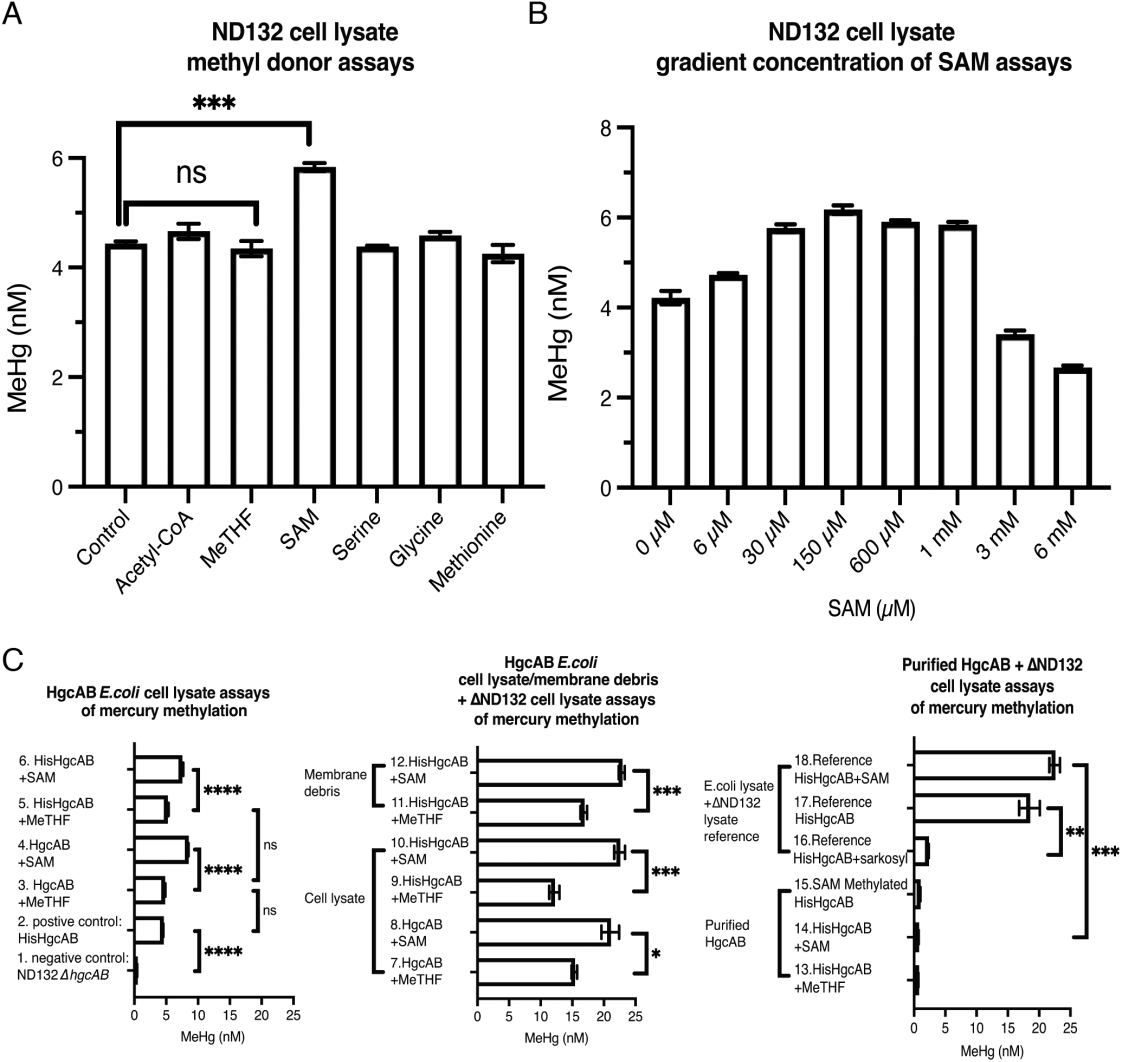
Hg methylation assays using *P. mercurii* ND132 cell lysate with different methyl donors and using *E. coli* BL21 cell lysates containing HgcAB. (*A*) Mercury methylation assays for *P. mercurii* ND132 cell lysate with various methyl donors. (*B*) Mercury methylation results for *P. mercurii* ND132 cell lysate with increasing concentrations of SAM. (*C*) The *Left* panel (rows 1 to 6) presents results of Hg methylation assays using *E. coli* BL21 cell lysates containing HgcAB or N-terminal His_6_-tagged HgcAB incubated with SAM or MeTHF. Row 1: negative control for Hg methylation assays using only ND132(∆*hgcAB*) cell lysate. Row 2: positive control for Hg methylation assays using *E. coli* BL21 cell lysates containing HgcAB. Rows 3, 5: *E. coli* BL21 cell lysates containing HgcAB or N-terminal His_6_-tagged HgcAB incubated with Me-THF. Rows 4, 6: Hg methylation assays performed as in rows 3, 5 except using SAM as methyl donor. The *Middle* panel presents results of Hg methylation assays using *E. coli* BL21 cell lysates containing HgcAB or N-terminal His_6_-tagged HgcAB + *P. mercurii* ND132 (∆*hgcAB*) cell lysates incubated with SAM or MeTHF. Rows 7 to 10: Hg methylation assays performed as in rows 3 to 6 but included *P. mercurii* ND132 (∆*hgcAB*) cell lysates. Row 11 to 12: Assays as in rows 9 and 10, respectively, except using *E. coli* BL21(DE3) membrane debris separate from *E. coli* BL21-expressed N-terminal His_6_-tagged HgcAB. The *Right* panel presents results of Hg methylation assays using purified N-terminal His_6_-tagged HgcAB and reference controls, including *E. coli* BL21 cell lysates + *P. mercurii* ND132 (∆*hgcAB*) cell lysates containing N-terminal His_6_-tagged HgcAB incubated ±SAM or ±Sarkosyl. Rows 13 to 14: Hg methylation assays with purified HgcAB and ND132(∆*hgcAB*) cell lysate with SAM/MeTHF. Row 15: SAM methylated purified HgcAB were incubated with ND132(∆*hgcAB*) cell lysate, and mercury methylation assays were performed. Row 16: *E. coli* BL21 cell lysates containing N-terminal His_6_-tag HgcAB were incubated with Sarkosyl and *P. mercurii* ND132 (∆*hgcAB*) cell lysates, and mercury methylation assays were performed. Row 17: Same as row 16, except with Sarkosyl. We conducted the assays described in rows 16 and 17 because we use Sarkosyl in our purification procedure, and these assays revealed that Sarkosyl disrupts the second half-reaction. Row 18: Same as Row 10. All *P* values were obtained through a *t* test using GraphPad Prism 9. Error bars represent SD. N = 3 for each bar. ns: *P* > 0.05; *P ≤ 0.05; **P ≤ 0.01; ***P ≤ 0.001; ****P ≤ 0.0001. Details of assay conditions are described in *SI Appendix*, Table S2. Experimental details are provided in the *Materials and Methods* and *SI Appendix*.

**Fig. 2.**
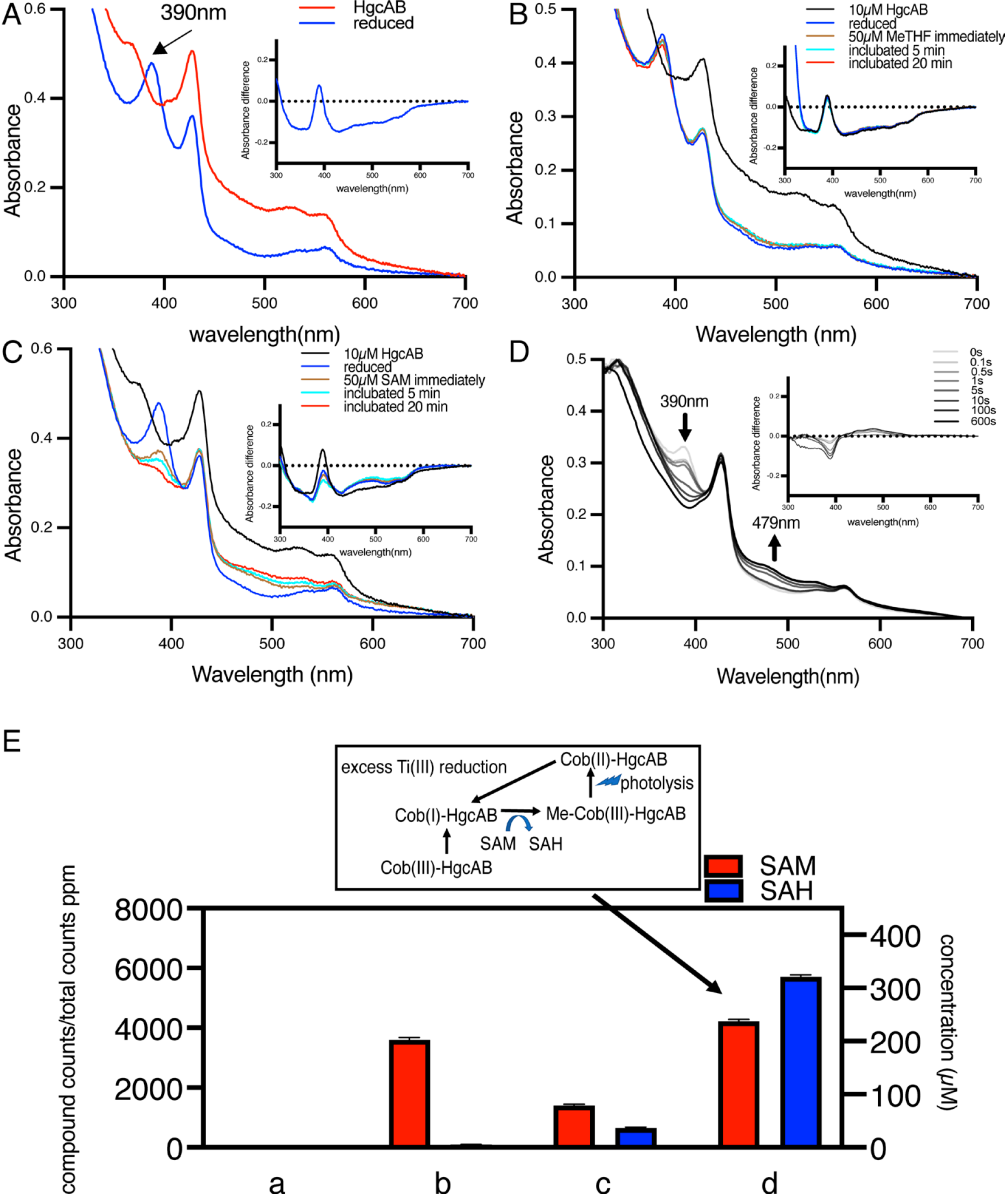
UV-visible spectroscopic and LC–MS characterization of HgcAB methylation. (*A*–*D*) UV-vis spectra of (*A*) 10 µM Cob(III)-HgcAB before and after addition of 100 µM Ti(III) citrate reductant to obtain Cob(I)-HgcAB. (*B*) 10 µM Cob(III)-HgcAB and after reduction to Cob(I)-HgcAB as in *A*; then, after addition of 50 µM Me-THF at 0, 5, and 20 min. (*C*) 10 µM Cob(I)-HgcAB formed as in *A* and at 0, 5, and 20 min after addition of 50 µM of SAM. (*D*) 5 µM Cob(I)-HgcAB after reaction with 50 µM of SAM for 0, 0.1, 0.5, 1, 5, 10, 100, and 600 s. *Insets* in each figure show the UV-vis spectral differences between reduced HgcAB or methylated HgcAB and initial HgcAB spectra [Cob(III)-HgcAB or Cob(I)-HgcAB]. (*E*) Triple quadruple time of flight liquid chromatography/mass spectrometric (Q-TOF LC/MS) identification of SAH as product of the SAM methylation reaction, as in “d”. (a) 50 µM Cob(I)-HgcAB, (b) quenched “a” + 200 µM SAM, (c) “a”+ 150 µM SAM, and (d) “a” + 400 µM SAM, under photolysis conditions to enable multiple turnovers as described in the scheme above the data and the main text. The *Y* axis represents SAM or SAH (ppm peak height from the LC–MS signal, and µM SAM remaining or SAH generated). Error bars represent the SD (N = 3). Other details are provided in the *Materials and Methods* and *SI Appendix*.

Prior studies have suggested that the methyl donor for mercury methylation is Me-THF (7, 12). However, enzymatic analyses of the overall mercury methylation reaction by cell lysates (Fig. 1) and enzymatic studies of the first half-reaction (cobalamin methylation) with the purified HgcAB complex (Fig. 2 *B*–*D*) do not support this hypothesis. Of the potential methyl donors tested, including Me-THF, acetyl coenzyme A (acetyl-CoA), serine, glycine, methionine, and SAM, only the inclusion of SAM in the assay led to a significant increase in MeHg formation above background levels in cell lysates (Fig. 1*A*). We also observed a positive correlation between SAM concentration and MeHg yield until the SAM concentration reached saturation around 150 µM (Fig. 1*B*). Cell lysate from BL21(DE3) expressing HgcAB exhibited increased Hg methylation activity (Fig. 1*C*) compared with the negative control (*Left* panel, rows 1 and 2). Importantly, inclusion of SAM instead of Me-THF produced significantly higher MeHg yields in *E. coli* lysates containing HgcAB (Fig. 1 *C*, *Left* panel, rows 3 vs. 4, 5 vs. 6), independent of whether HgcA contained (*Left* panel, rows 5 and 6) or lacked (*Left* panel, rows 3 and 4) an N-terminal His_6_-tag. Similar results were observed with *E. coli* lysates containing HgcAB mixed with Δ*hgcAB* ND132 cell lysate (Fig. 1 *C*, *Middle* panel, rows 7 vs. 8, 9 vs. 10 regardless of the His_6_-tag). Similar results were also obtained when the assays contained partially purified HgcAB instead of cell lysates (Fig. 1 *C*, *Middle* panel, rows 11 vs. 12).

The high background levels of MeHg in Fig. 1 are due to endogenous SAM in the cell lysates. Unfortunately, since SAM is such a crucial methyl donor involved in numerous cellular processes, we do not yet have a method to cleanly deplete SAM. However, even with the background level of MeHg, the addition of SAM resulted in a statistically significant increase in MeHg production. Including Me-THF (Fig. 1*A*, lane control and lane Me-THF) did not produce a statistically different yield of MeHg compared with controls. These data indicate that HgcA utilizes SAM, not Me-THF, for Hg methylation. We also can conclude that the N-terminal His_6_-tag on HgcA (Fig. 1 *C*, *Left* panel, rows 3 vs. 5) does not significantly affect the level of methylation. However, the overall Hg methylation activity is inhibited by Sarkosyl (Fig. 1 *C*, *Right* panel, rows 16 vs. 17), which was used during purification, as shown in cell lysate assays. This finding explains why the purified HgcAB complex does not exhibit mercury methylation activity in Δ*hgcAB* ND132 cell lysate (Fig. 1 *C*, *Right* panel, row 13 to 15). On the other hand, as shown in experiments with the purified protein, Sarkosyl does not affect the first half-reaction from Cob(I)- to Me-Cob(III)-HgcAB (Fig. 2*C*).

To further characterize the role of SAM as the methyl donor, we explored the formation of S-adenosyl-L-homocysteine (SAH), which should be formed in stoichiometric amounts relative to those of Cob(I)-HgcAB during methyl-Co(III) formation. Thus, we used LC–MS to separate and track the conversion of SAM (m/z = 399.14) to SAH (m/z of SAH is 385.13) during this single-turnover reaction (Fig. 2*E*). Upon addition of 150 µM SAM to 50 µM Cob(I)-HgcAB, about 50 µM SAH was formed, which equals the amount of HgcAB in the reaction (Fig. 2*E*, group c). SAH was not detected in the two control groups, when SAM was omitted and when the reaction was quenched before addition of SAM (Fig. 2*E*, group a and b). To convert this single-turnover to a multiple-turnover reaction, we reacted Cob(I)-HgcAB with excess SAM, while exposing the reaction to light from a fluorescent lamp. Under these conditions, the Me-Co(III) formed from reaction with SAM underwent photolysis to Co(II) followed by Ti(III)-dependent reduction to Cob(I), which reacted with the remaining SAM. Thus, this cycle will iteratively convert SAM to SAH (Fig. 2*E*, group d). Although MeHg is too toxic to perform multiple turnover HgcAB-catalyzed reactions between SAM and Hg^++^, these combined experiments demonstrate conclusively that SAM is the methyl donor to the cobalamin center of HgcAB to generate SAH and methylcob(III)alamin, which in the presence of Hg^++^ would then undergo demethylation to generate MeHg.

### Spectroscopic Characterization of the SAM-Dependent HgcAB Reaction.

**Fig. 3.**
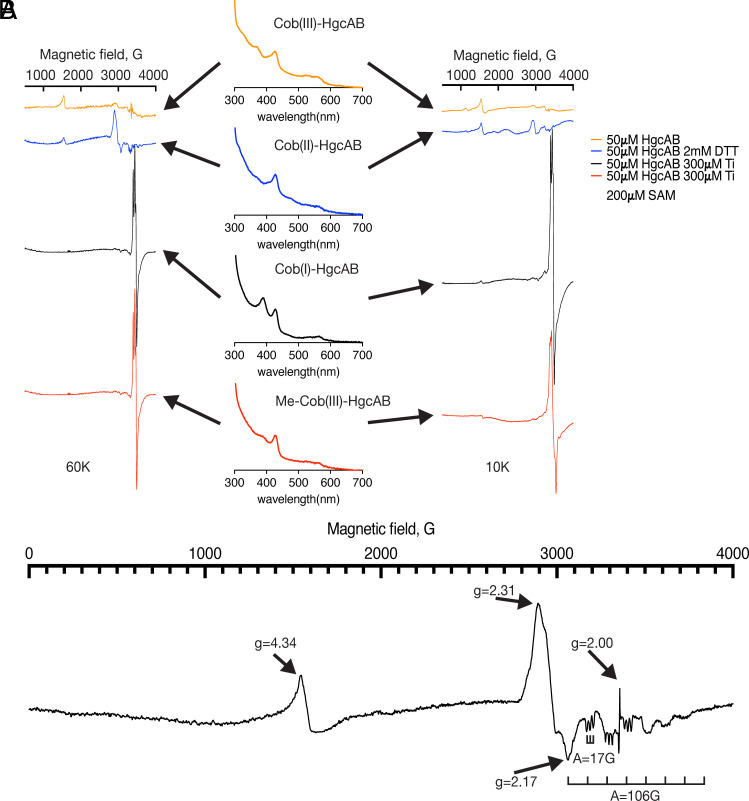
EPR spectroscopic characterization of the HgcAB methylation reaction. (*A*) EPR spectra at 60 K (*Left*) and 10 K (*Right*) of Cob(III)-HgcAB, Cob(II)-HgcAB, Cob(I)-HgcAB, and SAM-generated Me-Cob(III)-HgcAB. The middle figures show the UV-vis spectra associated with the corresponding EPR spectra on the *Left* and *Right*. (*B*) Detailed view of Cob(II)-HgcAB EPR spectrum, which indicates its base-on, Cys-off coordination mode. For further details, see the *Materials and Methods*.

**Fig. 4.**
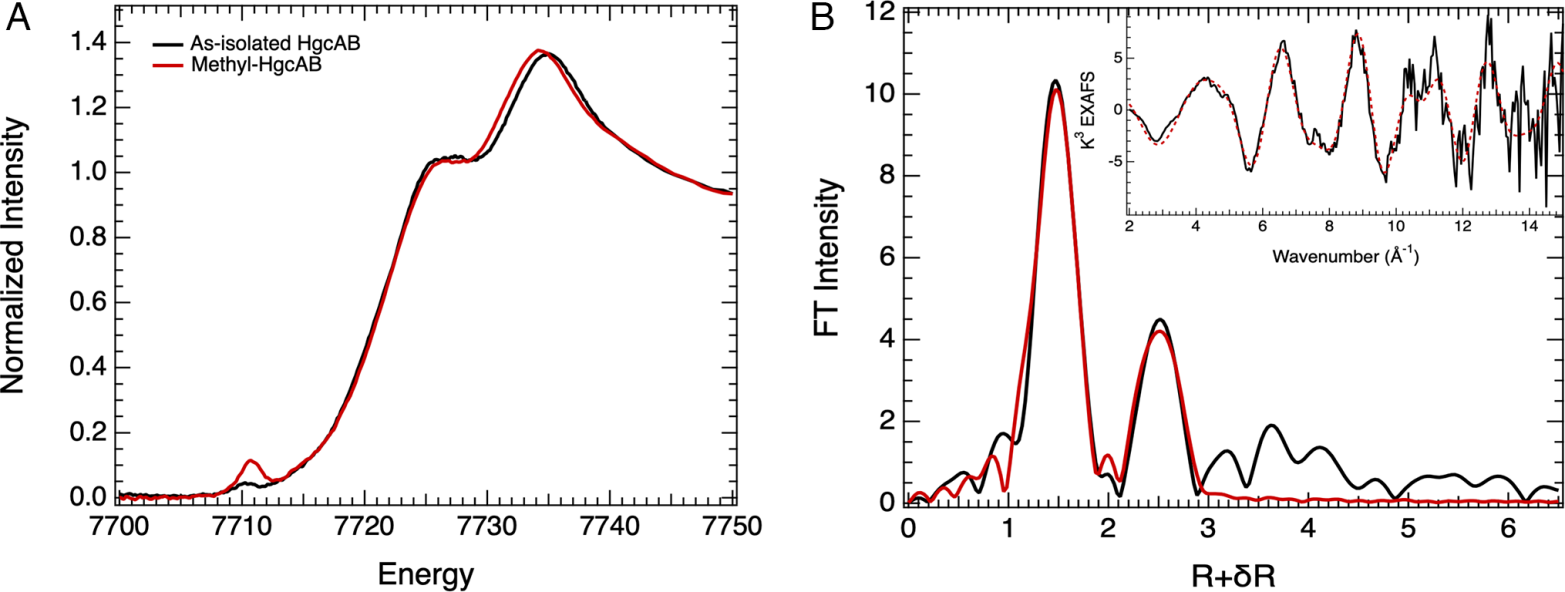
Co K-edge XAS of HgcAB. (*A*) Co K-edge XAS of methylated (red) and as-isolated (black) HgcAB. (*Inset*) Expanded Co K-pre-edge region. (*B*) Non-phase-shift-corrected Fourier transform and (*Inset*) corresponding EXAFS for methylated-HgcAB. Data (black), fit (red). For further details, see the *Materials and Methods* and *SI Appendix*.

With the purified protein complex, we spectroscopically and kinetically characterized only the first half-reaction (methylation of the Co center of HgcA), because potentially toxic levels of MeHg could have formed had we performed the full reaction. Thus, we monitored HgcAB methylation by single-turnover UV-visible (UV-Vis) (Fig. 2 *A*–*D*), EPR (Fig. 3 *A* and *B*), X-ray absorption (XAS) (Fig. 4) spectroscopies and transient stopped-flow kinetics (*SI Appendix*, Fig. S8). Upon treatment of the purified HgcAB complex (red, Fig. 2*A*; black, Fig. 2 *B* and *C*) with a ninefold excess of Ti(III)-citrate (Fig. 2 *B* and *C*), the characteristic 390 nm peak of cob(I)alamin slowly appeared (36). Addition of ninefold excess Me-THF in the dark did not cause any obvious UV-vis spectral changes even after 20 min (Fig. 2*B*). However, upon addition of a ninefold excess of SAM in the dark, the 390 nm Co(I) peak disappeared within ~20 s (Fig. 2*C*), as a broad 479 nm peak (Fig. 2*D*) appeared, indicating the consumption of cob(I)alamin and formation of methylcob(III)alamin. This reaction occurred >50-fold faster than SAM methylation of free cob(I)alamin, which takes ~20 min under these conditions (*SI Appendix*, Fig. S5).

In addition to the cobalamin spectra, we observed a 420 nm peak whose intensity varied slightly among different purification batches. We conducted pyridine hemochromagen and TMBZ heme gel staining assays (37), confirming the presence of a low level (10 to 15%) of heme copurifying with the HgcAB complex. As shown in the difference spectra in the Fig. 2, *Insets*, there is no change in the Soret band intensity or position during the HgcAB SAM methylation assays, suggesting that the heme does not play a role in Hg methylation by HgcAB and that it may merely be a contaminant of protein expression in *E. coli*. The heme appears to be tightly bound and could not be removed from the HgcAB complex by incubation with apo-myoglobin; furthermore, heme addition to the purified protein did not increase its levels. Additional studies are needed to firmly delineate any potential role of this heme ligand in Hg methylation.

**Fig. 5.**
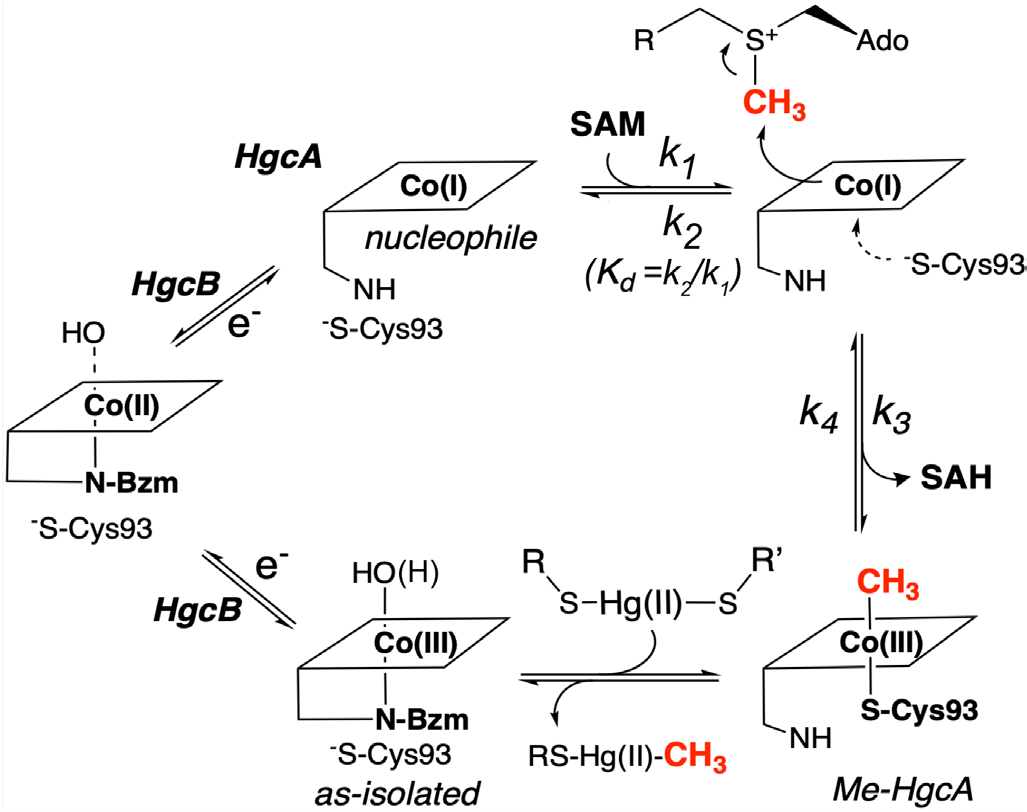
Proposed mechanism for enzymatic mercury methylation catalyzed by HgcAB. For the as-isolated Co(III) state, the upper axial ligand is likely to be water or OH. Two-electron reduction by the two ferredoxin-like [Fe_4_S_4_]^1+^ clusters of HgcB yields the nucleophilic Co(I) state, which attacks the electrophilic methyl group of SAM to generate methyl-Co(III)-thiolato cobalamin, which likely transfers its methyl group as a methyl radical (or methyl carbanion) to Hg(II) to generate MeHg.

To characterize the electronic states of B_12_ in HgcA during SAM-dependent methylation, we performed EPR spectroscopy at 60 K, which selectively visualizes cob(II)alamin, since the [4Fe-4S]^1+^ clusters relax too quickly to be observed above 20 K (38). Anaerobically purified HgcAB is EPR silent; however, after reduction by 2 mM DTT, an eight-line cob(II)alamin (S = 7/2, A_ll_ = 106 G) EPR signal appeared with a characteristic three-line superhyperfine pattern (A_N_= 17G) (Fig. 3 *A* and *B*), indicating axial N-coordination to the cob(II)alamin. These spectral characteristics resemble those of methionine synthase (39, 40) and other base-on (or His-on) cob(II)alamins, instead of base-off/His-off systems like the *Moorella thermoacetica* corrinoid iron–sulfur protein (CoFeSP) (41) or Cys-ligated cob(II)alamin, which we expected based on prior modeling results (14). The EPR spectra of Ti(III)-reduced HgcAB (both before and after addition of SAM) include a strong EPR signal at 3,400 G reflecting the excess Ti citrate; however, neither of these spectra contain features of cob(II)alamin, suggesting the presence of cob(I)alamin and methylcob(III)alamin, respectively, in these samples. Based on the EPR and UV-visible experiments, it appears that as-isolated HgcA contains cob(III)alamin (with a sulfur or nitrogen ligand), which undergoes mild reduction to an N-coordinated cob(II)alamin (Fig. 5). This state could appear as a catalytic intermediate if the methyl group transfers to Hg^++^ as a radical; on the other hand, Co(II) may only appear as an inactive species formed by oxidation of Co(I), as also observed in other cobalamin-dependent methyltransferases, which require reductive activation to reenter the catalytic cycle (42). The as-isolated or Co(II) states undergo low-potential reduction to cob(I)alamin, which is activated for SAM-dependent methylation to form methylcob(III)alamin.

To further examine the redox and ligation states of the metallocofactors in HgcAB, we performed XAS studies. Co K-edge XAS were measured on the as-isolated and methylated (Me-HgcAB) forms of HgcAB (Fig. 4*A*). The rising-edge energies (measured at 0.5 normalized intensity) are similar for both species and agree with previously reported cob(III)alamin species (43). The *Inset* of Fig. 4*A* shows the expanded pre-edge region, which represents the Co 1s → 3d transition and gains intensity through symmetry-allowed Co 3d-4p mixing. The pre-edge intensity of as-isolated HgcAB is characteristic of a typical octahedral Co(III) complex and supports the presence of weak axial ligands. Voigt fits reveal a 0.5 eV shift in energy and a ~3.7-fold increase in intensity in this feature upon methylation (Table 1). This increase is characteristic of a short axial Co-C bond, which leads to distortion in Co centrosymmetry and an increase in 3d-4p mixing. In related systems, the absolute intensity of this feature is proportional to the Co-C bond distance. *SI Appendix*, Fig. S6*A* and its *Inset* compare Me-HgcAB with methyl-cob(III)alamin (Me-Cbl) and methyl-co(III)binamide (Me-Cbi). Me-Cbl is in the base-on conformation, with a trans-axial Co-N_DMB_ ligand, while Me-Cbi lacks a tightly bound trans-axial ligand, which significantly increases the pre-edge intensity. The similarity in pre-edge intensity with Me-Cbl indicates the presence of a trans-axial ligand in Me-HgcAB.

**Table 1.** DFT and TD-DFT geometry optimization calculations on the Me-Co(III) states of HgcAB, methylcobalamin, and aquocobalamin related to the XAS data described in Fig. 4 and *SI Appendix*, Fig. S6^*^

	Methyl-Cob(III)-Cys	Methylcobalamin	Aquocobalamin
Co-N(Corrin)	1.924^*^	1.914^*^	1.883^*^
	1.875	1.873	1.926
	1.876	1.871	1.920
	1.936	1.922	1.883
Average Co-N (corrin)	1.903	1.895	1.903
Coaxial ligand (methyl or water)	2.007	1.974	2.050
Co-trans-axial ligand (S, DMB, water)	2.469	2.153	1.916

^*^All distances in Å.

Best fits to the non-phase-shift-corrected Fourier transforms and Extended X-ray Absorption Fine Structure (EXAFS) data of Me-HgcAB using Feff (Fig. 4*B* and Table 1) reveal the presence of Co-C coordination at 2.02 Å and a long Co-S contribution at 2.5 Å. Me-Cbl fits, performed as a reference for Me-HgcAB, (Table 1 and *SI Appendix*, Fig. S6*B*) are consistent with a shorter Co-C bond at 1.98 Å and a Co-N contribution at 2.16 Å. A Co-S interaction similar to Me-HgcAB was observed in a different cobalamin cofactor (44), although the species was not methylated. These data reveal the formation of a trans-axial Co-S(Cys) bond in a methylated cobalamin cofactor. EXAFS data for the as-isolated species were obtained to lower k and were well modeled with a six-coordinate species with two axial Co-N/O ligands at 2.39 Å (*SI Appendix*, Fig. S6*C*). DFT and TD-DFT calculations were performed on a six-coordinated model with N(DMB)/OH_2_ axial coordination (Table 1), which structurally reproduce the EXAFS data. Co K-pre-edge TD-DFT calculations were also consistent with the experimental data (*SI Appendix*, Fig. S6*D*).

DFT geometry optimization of a methylated Cob(III) species with an axially bound S(Cys) yields a Co-C distance of 2.01 Å and a Co-S at 2.47 Å (Table 1 and *SI Appendix*, Fig. S6*B*), consistent with the EXAFS results. Furthermore, DFT reveals that the replacement of N_DMB_ by S(Cys) results in an elongation of the Co-methyl bond by ~0.03 Å. This elongation contributes to the decrease in the XANES pre-edge intensity of the Me-HgcAB relative to Me-Cbl (*SI Appendix*, Fig. S6 *A*, *Inset*), owing to a decrease in Co 4p character in the states that contribute to the pre-edge feature in Me-HgcAB, which is reproduced well by TD-DFT calculations (*SI Appendix*, Fig. S6*E*). These results indicate the presence of a long Co-S bond, supporting a Co-S(cys) coordination in Me-HgcAB. The difference in Co-C bond length relative to Me-Cbl suggests that the unprecedented Co-S interaction in a methylated cobalamin cofactor might be designed to tune the Co-C bond and poise it for Hg methylation.

The Co XAS data of the Cys-on methylated state are consistent with a prior study by Li et al. who observed a Co-S(Cys) bond at a distance of 2.56 Å in the base-off, unmethylated B_12_ trafficking protein CblD (44). This is an experimental validation of a Co-S assignment originally predicted in 2013 (3) and later supported by quantum chemical calculations performed by us (24) and by others (23) as well as using site-directed mutagenesis and in vivo Hg methylation experiments (3, 18). These latter experiments described the critical roles of Cys93 in HgcA and Cys73, Cys94, and Cys95 in HgcB for Hg methylation. It is important in future investigations to determine whether the Co-S bond is most important for methylation of Co in the first half-reaction or for Hg in the second half-reaction.

### Kinetic Studies of the SAM-Dependent Methylation of B_12_ by HgcAB.

We propose that the first half-reaction, generation of methyl-Co(III), involves transfer of a methyl cation (Fig. 5). We also suggest that the second half-reaction is likely linked to a unique radical- or anion-based mechanism that may involve the conserved C-terminal cysteines (C94 and 95) in HgcB, for Hg transfer from solution and/or for reaction with the Co-S center (involving Cys93) to yield MeHg. Cobalamin methylation by HgcAB occurs 50 times faster than the analogous SAM-dependent methylation of TrsM, GenK, Fom3, and PhpK, and of free Cob(I)alamin (45–49); thus, we characterized the kinetics via stopped-flow UV-vis spectroscopy. We mixed 10 µM Ti(III) citrate-reduced HgcAB with increasing concentrations of SAM and observed that decay of the 390 nm Co(I) absorption follows a double exponential progress curve, matching the increase at 479 nm (*SI Appendix*, Fig. S7). The stopped-flow data were acquired on a logarithmic time scale and analyzed by fitting the data to a double exponential equation (Eq. **2**) for both 390 nm and 479 nm (*SI Appendix*, Fig. S7), yielding rate constants *k_fast_* and *k_slow_* (Eq. **2**) (*SI Appendix*, Table S4), which correspond to the fast and slow changes in the 390 nm and 479 nm signals. When *k_fast_* or *k_slow_* were plotted against SAM concentration, a saturation curve was observed for *k_fast_* (*SI Appendix*, Fig. S8*D*), while *k_slow_* remained SAM independent. Additionally, a comparison of the UV-vis difference spectra associated with *k_slow_* or *k_fast_* revealed significant differences (*SI Appendix*, Fig. S9). The amplitude of the 390 nm peak for *k_fast_* accounted for 75% of the total and was associated with the increase at 479 nm. Conversely, the broad shoulder increase spanning from 450 nm to 520 nm was associated with *k_slow_*. These observations suggest that *k_slow_* corresponds to a side reaction(s), likely unrelated to Cob(I) methylation (*SI Appendix*, *Supplementary Materials* and Figs. S7 and S9). These side reaction(s) may involve nonenzymatic oxidation of Co(I) or slow homolytic cleavage of methyl-Co(III) to generate Co(II). Thus, we focused our attention on *k_fast_*.

We propose a two-step reversible mechanism described by Eq. **1** (*K_d_* = *k_2_*/*k_1_*). The traditional nonlinear fitting approach for acquiring kinetic parameters involves many approximations, such as square root approximations and other hypothetical assumptions, to simplify complex equations that are difficult to fit. These approximations can introduce errors, resulting in kinetic parameters that deviate significantly from the true values. Using simulation and fitting through kinetics software is an effective way to mitigate this issue. Therefore, we built a double-step reversible mechanism model in KinTek Explorer software and fitted our kinetic data with the model to acquire accurate kinetic parameters. To prevent interference from nonenzymatic oxidation of Co(I) or the slow homolytic cleavage of methyl-Co(III) generating Co(II), we selected 390 nm and 479 nm kinetics data within the 0.05 s to 5 s range. The *k_1_* and *k_2_* values for the forward and reverse reactions of the first step are 0.55 ± 0.04 µM^−1^·S^−1^ and 16 ± 1 s^−1^, and the *k_3_* and *k_4_* values for the forward and reverse reactions of the second step are 0.160 ± 0.003 s^−1^ and 0.002 ± 0.001 µM^−1^·s^−1^, according to the 390 nm kinetics data (*SI Appendix*, Fig. S8*A*). When fitting the model using the 479 nm data, *k_1_*, *k_2_*, *k_3_*, and *k_4_* are 0.40 ± 0.09 µM^−1^·s^−1^, 16 ± 4 s^−1^, 0.13 ± 0.02 s^−1^, and 0.002 ± 0.01 µM^−1^·s^−1^, respectively (*SI Appendix*, Fig. S8*B*). The global fitting, which includes both 390 nm and 479 nm kinetics data, yielded values of *k_1_*, *k_2_*, *k_3_*, and *k_4_* of 0.48 ± 0.09 µM^−1^ï·s^−1^, 16 ± 3 s^−1^, 0.15 ± 0.02 s^−1^, and 0.002 ± 0.009 µM^−1^·s^−1^ (*SI Appendix*, Fig. S8*C*). K_D_(SAM) thus equals 33 µM. The 50 to 75-fold larger rate of SAH formation (*k*_3_) relative to that of reformation of SAM (*k_4_*), indicates that the SAM-dependent methylation of HgcA is irreversible.


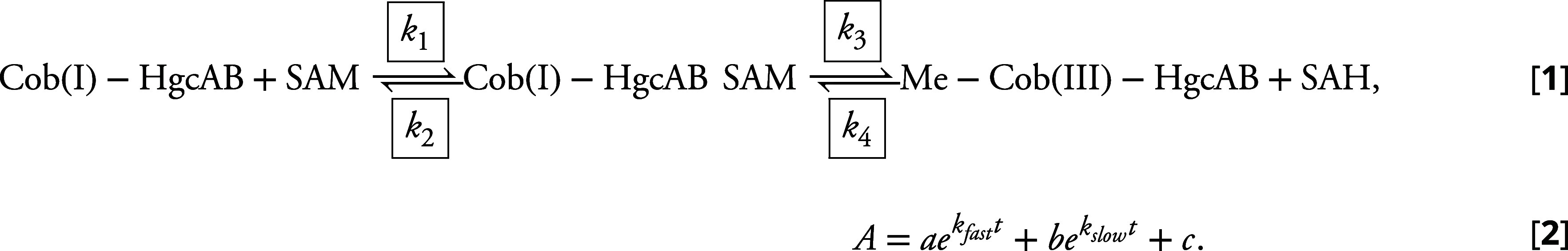


## Discussion

It has been three decades since the discovery of the enzymatic basis for microbial mercury methylation. Previous mercury methylation studies using isotope labeling mass spectrometry identified an association between the Wood–Ljungdahl pathway and mercury methylation, suggesting Me-THF as the methyl donor (11–13). Subsequent sequence analyses reinforced this hypothesis because of the homology between the CoFeSP involved in acetyl CoA synthesis and the cobalamin binding domain of HgcA (3). Furthermore, metabolic studies and sequence comparisons implicated Me-THF as the methyl donor for mercury methylation; however, no direct evidence for these assertions has been reported due in part to the low levels of HgcAB expression in microorganisms and the difficulties of purifying this O_2_-sensitive, membrane-associated protein complex.

Here, we propose a mechanism for HgcAB-dependent mercury methylation that uses SAM as a methyl donor (Fig. 5). Based on our spectroscopic studies, the resting state of the mercury methylation complex is Cob(III)-HgcAB. It is unclear whether the upper axial ligand is water or OH^−^, and the p*K*_a_ for aquo-Cob(III)-alamin is in the pH 6-8 range. Correlation of the pre-edge XAS spectra with TD-DFT agrees with this assignment, indicating a tetragonally elongated Cob(III) species with weakly bound water or OH. Since Cob(III) is low-spin *d_6_*, such a distortion would yield vacant dx^2^-y^2^ and d_z_^2^ states, which can be reduced by a weak reductant such as dithiothreitol (DTT) and form an EPR-active N-coordinated Cob(II)-HgcAB. The octet Co(II) and triplet N-splittings are similar in magnitude to those seen in the base-on states of methionine synthase (42), reflecting either Co(II) coordination of the N-benzimidazole attached to the cobalamin ring or perhaps a His residue. Based on our prior modeled structure of HgcA, there are no nearby His residues (14), favoring benzimidazole as the axial ligand; however, further studies will be required to identify the axial Co(II) ligand.

During the catalytic cycle, HgcAB is reduced to the Cob(I)-HgcAB state through electron transfer mediated by the [4Fe-4S] clusters in HgcB. These two clusters are unlikely to function in 5-deoxyadenosyl radical generation like class B radical SAM enzymes, which only coordinate with three cysteine ligands (50). In these site-differentiated [4Fe-4S] clusters, the differentiated iron does not coordinate with cysteine and can interact with SAM to generate a 5-deoxyadenosyl radical. This radical then initiates catalysis by substrate H atom abstraction at the eventual methylated position, with the methyl group originating from another molecule of SAM via intermediate methylation of the cobalamin cofactor. Instead, based on sequence analysis and structural modeling, both [4Fe-4S] clusters in HgcB have four associated cysteine ligands (3, 14), homologous to typical 8-Fe ferredoxins (51, 52). Thus, we propose that both [4Fe-4S] clusters in HgcB serve as electron transfer mediators. These two [4Fe-4S] clusters in HgcB exhibit complex EPR spectra when they are both reduced (*SI Appendix*, Fig. S4), indicating they are spaced close enough (<14 Å) to facilitate rapid electron transfer to regenerate Co(I) during reactive activation and catalysis. The origin of these two reducing equivalents is unknown. We suggest that pyruvate-ferredoxin oxidoreductase, hydrogenase, or other low-potential electron donors could serve this role.

After two-electron reduction to Cob(I)-HgcAB, a molecule of SAM can bind to the HgcAB complex and transfer its methyl group to Cob(I)-HgcA as a methyl cation with the generation of one molecule of SAH. The Cys thiolate ligand then coordinates as a lower axial ligand to stabilize Me-Cob(III)-HgcAB. Then, Hg^++^ is delivered as Hg(RS)_2_ to the active center of HgcAB and the methyl group from Me-Cob(III)-HgcAB is transferred to Hg(RS)_2_ as a methyl radical or carbanion, forming MeHgSR and another thiolate (RS^−^), reverting HgcAB back to its Cob(III)-HgcAB resting state.

Questions about the mechanistic details of the second half-reaction remain. Conducting the full Hg methylation assay with purified, active enzyme will generate large quantities of neurotoxic methylmercury. It is also unknown whether the enzyme can accept monomethylmercury as a substrate, and potentially generate the even more deadly dimethylmercury (53). Figuring out how to safely conduct this experiment, or validating some alternative less dangerous assay, will be a significant future undertaking.

Our findings conclusively demonstrate that SAM, not Me-THF, serves as the source of the methyl group in MeHg, further expanding the biochemical role of SAM. The understanding of SAM-dependent enzymes already has undergone a major expansion over the past two decades, with the discovery of radical SAM enzymes (54). A variety of SAM- and cobalamin-dependent reactions are catalyzed by class B radical SAM enzymes; examples include TokK, Fom3 (45, 55–57), and Mmp10 (58–60). TsrM, which catalyzes methylation of posttranslationally modified peptides (RiPPs) is an atypical member of this class in that it does not appear to use a dAdo radical, as we propose for HgcAB (36, 49). Additionally, we are not aware of any other class B radical SAM enzyme and corrinoid iron–sulfur methyltransferase that contains an essential transmembrane domain. The involvement of SAM in HgcAB-catalyzed Hg methylation also distinguishes HgcAB from the CoFeSP involved in B_12_ and Ni ion methylation in the Wood–Ljungdahl pathway.

In summary, demonstrating SAM as the methyl donor in MeHg formation by the HgcAB enzymatic system overturns a paradigm linking this process to Me-THF. MeHg perversely biomagnifies through the food web to become a potent human neurotoxin. Significant challenges of low expression levels and poor cofactor incorporation were overcome to allow enzymatic and biophysical characterization of this integral membrane-associated B_12_- and Fe_4_S_4_-dependent system. Unusual features of the HgcA active site include formation of a Me-B_12_ intermediate with a cysteine thiolate serving as a lower axial cobalt ligand. These findings may promote development of strategies to mitigate against the microbial production of toxic Me- and diMe-Hg in the environment.

## Materials and Methods

If not specified, all chemicals were commercially purchased from Sigma-Aldrich, St. Louis, MO. In all assays of the first half-reaction of HgcAB (methylation of the cobalamin in HgcA), we were cautious to ensure that no forms of Hg are present. Full Experimental Details for protocols and reagents related to the Materials and Methods are provided in *SI Appendix*.

### Cloning, Expression, and Purification of HgcAB.

To overcome the low native protein levels, we cloned the *hgcA* and *hgcB* genes from *P. mercurii* (formerly *D. desulfuricans ND132*) in tandem into *E. coli* pETDuet-1 expression vector (Novagen, Madison, WI). Sequences were verified by Sanger sequencing (University of Michigan DNA Sequencing Core). When expressed independently of HgcB, HgcA was unstable and insoluble, lacked cobalamin, and had very low expression levels. Similarly, HgcB expressed without HgcA was insoluble. However, expression of HgcA and HgcB in the tandem cloning sites of the pET duet vector produced a soluble HgcAB complex.

To ensure that the two [4Fe-4S] clusters were incorporated into HgcB and the cobalamin center into HgcA, we cotransformed the pRKisc vector for [4Fe-4S] cluster synthesis (29–31) and the pBtu vector for cobalamin uptake (32) with the HgcAB-containing pETDuet-hgcA-hgcB or pETDuet-His-hgcA-hgcB vector into both *E. coli* BL21(DE3) and C41(DE3) strains (28). In cell lysate assays for mercury methylation, constructs cloned into BL21(DE3) exhibited mercury methylation activity, while those cloned into C41(DE3) did not (not shown). Thus, we used the BL21(DE3) strain as our expression platform. After performing parallel experiments in BL21(DE3) under different conditions, we observed that the N-terminal His-tag does not significantly influence the HgcAB mercury methylation activity (compare rows 3 & 5 in Fig. 1*C*).

Anaerobic expression of HgcAB in M9-ETA media (components listed in *SI Appendix*, Table S1) provided the highest activity (*SI Appendix*, Fig. S1 and Table S3). Thus, we selected this growth condition to express the N-terminal His-tagged HgcA with a nontagged HgcB for further purification and characterization. We used a 10 L fermenter for HgcAB expression. Use of an N-terminal His_6_-tag on HgcA enabled purification of the HgcAB complex using Ni-NTA agarose chromatography (33). Because HgcA is a transmembrane protein, detergents were used to maintain solubility of the HgcAB complex. Our optimized purification protocol included 0.3% Sarkosyl and 0.6% Triton X-100, which enabled elution of homogeneous HgcAB from the Ni-NTA agarose.

All purification steps were performed in an anaerobic chamber that maintained O_2_ levels below 1 ppm. The cell pellets obtained from fermentation were resuspended in anaerobic Buffer A (50 mM K_2_HPO_4_, 100 mM NaCl, 10% glycerol, and 1 mM TCEP, pH 7.4) containing protease inhibitor and 10 mM imidazole (10 mL buffer/g cell pellets). Next, the resuspended cell pellets were sonicated and the cell lysates were anaerobically centrifuged. The supernatants were discarded, and the precipitants containing the membrane debris were weighed and resuspended (0.13 g/mL) in Buffer A with 1% Sarkosyl, 2% Triton X-100, 10 mM imidazole for 1 h at room temperature and sonicated, resuspended, and resonicated. The sonicated mixture was anaerobically centrifugated and the supernatant was applied to a Ni-NTA agarose column pre-equilibrated with Buffer A containing 0.3% Sarkosyl, 0.6% Triton X-100, washed, and eluted with Buffer A containing 0.15% Sarkosyl, 0.15% Triton X-100, 250 mM imidazole. The resulting HgcAB complex was typically >95% pure, containing the expected 37 kDa HgcA and the 11 kDa HgcB Coomassie-stained protein bands by SDS-PAGE (*SI Appendix*, Fig. S2).

The HgcAB complex was buffer exchanged using an Amicon centrifugal filter unit (Amicon® Ultra - 0.5 mL 30 kDa, Amicon, Miami, FL) and dialyzed overnight against Buffer B (20 mM HEPES, 150 mM NaCl, 1 mM TCEP, and 10% glycerol, pH 7.4). To prepare HgcAB samples for XAS characterization, an additional ethylenediaminetetraacetic acid (EDTA) dialysis step was included to remove Ni contamination that interferences XAS signal. After buffer exchange using an Amicon centrifugal filter unit, HgcAB samples were dialyzed overnight against Buffer C (20 mM HEPES, 150 mM NaCl, 1 mM TCEP, 1 mM EDTA, 0.1% Sarkosyl, and 10% glycerol, pH 7.4). Then, the HgcAB samples were dialyzed overnight against Buffer B (20 mM HEPES, 150 mM NaCl, 1 mM TCEP, and 10% glycerol, pH 7.4). The purified HgcAB complex stoichiometrically bound cobalamin (34, 35) (*SI Appendix*, Fig. S3 *A* and *B*) and two [4Fe-4S] clusters. MS/MS identification of SDS-PAGE gel slices validating the identity of the 37 and 11 kDa bands (*SI Appendix*, Fig. S2), and purified protein provided ~90% coverage for both HgcA and HgcB (*SI Appendix*, Fig. S3*C*).

### *P. mercurii* Culture Conditions and Preparation of Cell Lysates.

WT *P. mercurii* or Δ*hgcAB* cultures (ND132 cells lacking the *hgcA* and *hgcB* genes) were prepared as described previously (25) and were grown in modified minimal organic basal medium with yeast extract (MOY) (18) containing 40 mM fumarate, 40 mM pyruvate, and 1.2 mM thioglycolate and 1 mM cysteine·HCl as reducing agents for approximately 3 d (OD_600_ = 0.3) at 32 °C under anaerobic conditions. Cell pellets were harvested at 4 °C by centrifugation for 20 min at 7,500 × g in centrifuge bottles with sealing closures. Then, the cell pellets were washed two times with deoxygenated phosphate-buffered saline (PBS), pH 7.0, and resuspended in cold lysis buffer containing Pierce™ EDTA-free protease inhibitor and 2 mM DTT in PBS, pH 7.0, and disrupted on ice by sonication under strictly anaerobic conditions inside a glove box under an N_2_ atmosphere (O_2_ ≤ 1 ppm) with minimal exposure to ambient light. Unlysed cells were removed by centrifugation at 30,000 × g for 1 h at 4 °C. Total protein concentration of cell lysates was determined by the Bradford method with bovine serum albumin (BSA) (Millipore Sigma, St. Louis, MO) as standard and the lysates were stored in aliquots at −80 °C in amber glass vials with PTFE-lined silicone caps.

### HgcAB Analytical Assays.

Protein was determined using the BCA assay based on a standard curve generated by BSA. The cobalamin content of HgcAB was determined as the dicyano-Co(III)balamin complex by treatment of the protein solution with KCN at 95 °C (61) and measuring the dicyano-cob(III)alamin concentration by UV-visible spectroscopy using extinction coefficients of 26,200, 7,400, and 8,600 M^−1^cm^−1^ at 367, 540, and 580 nm (34, 35). Representative spectra of as-purified HgcAB and derivatized dicyano-cob(III)alamin are shown in *SI Appendix*, Fig. S3 *A* and *B*, respectively. The HgcAB [4Fe-4S] cluster occupancy was determined by EPR after reduction by sodium dithionite (1 mM, final) for 2 h at room temperature. The EPR spectrum (*SI Appendix*, Fig. S4) indicated a coupled [4Fe-4S]^+^ system, consistent with a classic eight iron ferredoxin (51, 52). The spin concentration was measured by double integration referenced to a 1 mM copper perchloride standard.

### Mercury Methylation Cell Lysate Assays.

These experiments were designed to produce <50 pmol of MeHg and performed in special facilities at ORNL to ensure safety. Cell lysates of *E. coli* strains containing the overexpressed HgcAB complex were prepared from frozen washed cell pellets after resuspension in lysis buffer and sonication under anaerobic conditions, as described above. After removal of unlysed cells by centrifugation and determination of the total protein concentration. Hg methylation assays were conducted in an anaerobic glove box under a pure N_2_ atmosphere ([O_2_] < 1 ppm) in the dark. Samples were added to 4 mL amber glass vials and the total protein concentration was adjusted with anoxic PBS buffer to 1.5 mg/mL. For experiments in which cell lysates were mixed, aliquots of the *E. coli* lysates were added to a *P. mercurii* Δ*hgcAB* cell lysate, to a protein concentration of 0.1 mg/mL unless specified otherwise.

To study the effect of metabolites on Hg methylation in ND132 cell lysates, freshly prepared stocks (1.5 mM) of 5-Me-THF, S-adenosyl methionine (SAM), acetyl-CoA, L-glycine, L-methionine, or L-serine were added to Hg methylation assays at a final concentration of 30 µM and in a final volume of 1 mL, or at increasing concentrations in establishing the *K_m_* for SAM.

In all mercury methylation assays, samples were equilibrated for 5 min before adding an aliquot of a freshly prepared HgCl_2_ stock solution (1.5 µM) to a final concentration of 30 nM and incubated at 32 °C for 2 h, quenched by adding 0.5% (v/v) trace-metal grade H_2_SO_4_, and stored at −20 °C until MeHg was analyzed by inductively coupled plasma mass spectrometry (ICP-MS). MeHg was measured using a modified EPA Method 1630 via distillation and ethylation (3, 62). Modifications to the EPA methods included the use of isotope dilution with enriched stable isotopes to determine total Hg and methylmercury concentrations, and detection of Hg by ICP-MS to separate the various Hg isotopes (63).

### Single Turnover Assays of HgcAB Methylation.

To track the HgcAB-catalyzed cobalamin methylation reaction, we performed the enzymatic assay in Buffer B. A titanium(III) citrate (Ti(III) citrate) solution was prepared by adding 100 µL of 15% Ti(III) chloride into a premixed buffer containing 1,125 µL of 0.5 M sodium citrate and 400 µL of 1 M Tris buffer, pH 8.0 for 10 min at room temperature. An aliquot was diluted into Buffer B to 66 mM Ti(III) citrate for cobalamin reduction.

Methylation assays were performed using 10 µM HgcAB in Buffer B. Then, 100 µM Ti(III) citrate (final) was added to reduce the as-purified Cob(III)- to Cob(I)-HgcAB and appearance of the 390 nm Co(I) peak was monitored. Next, 50 µM methyl donor was added and disappearance of the 390 nm Co(I) peak and appearance of a broad 479 nm peak were monitored.

The nonenzymatic methylation of cobalamin was performed similarly using 10 µM reduced hydroxocobalamin in Buffer B. To thoroughly reduce the 10 µM of hydroxocobalamin, 400 µM Ti(III) citrate was added for a 20 min reaction, and reduction was monitored by appearance of the 390 nm Co(I) peak in the UV-vis spectrum. Meanwhile, the excess Ti(III) citrate significantly increased the baseline absorbance (*SI Appendix*, Fig. S5*A*). Then, 50 µM SAM was added and the 390 nm Co(I) peak slowly disappeared in 20 min, concomitant with the appearance of a broad 520 nm peak (*SI Appendix*, Fig. S5*B*).

### HgcAB Stopped-Flow Kinetic Assays.

To characterize the kinetics for the HgcAB methylation reaction, stopped-flow UV-vis experiments were performed at 21 °C within the anaerobic glove box on an Applied Photophysics (Leatherhead, England). spectrophotometer equipped with monochromator and photodiode array (PDA) detector. In one syringe (Reagent A), 10 µM HgcAB in Buffer A was reduced by addition of 100 µM Ti(III) citrate (final). The other syringe (Reagent B) contained SAM (100 µM, 200 µM, 400 µM, 800 µM, 1,200 µM, 1,600 µM, or 2,000 µM) in the same buffer. Reagents A and B were mixed via a 1:1 ratio in single-mixing mode and the experiments were performed in triplicate. UV-vis spectra were collected from 300 nm to 700 nm for analysis and fitting. An example is shown in Fig. 2*D*, in which data were acquired at 50 µM SAM after mixing and spectra from different time points of the reaction were superimposed.

The stopped-flow data were acquired using Pro-data SX software. Each set of reactions was monitored for 600 s and 1,000 spectra were collected in a logarithmic time interval. Analysis was performed using Pro-data viewer software and data at both 390 nm and 479 nm were fit to a double exponential equation (*SI Appendix*, Fig. S7) to yield both *k_fast_* and *k_slow_* (*SI Appendix*, Table S4). The fitting of *k_fast_* obtained from both 390 nm and 479 nm versus SAM concentration was performed by in GraphPad Prism 9 (*SI Appendix*, Fig. S8*D*). The kinetic data from 0.05 to 5 s at 390 nm and 479 nm were fit to a two-step reversible model via KinTek Explorer 11.1.0 software, to obtain the rate constants *k_1_*, *k_2_*, *k_3_*, and *k_4_*. The fitting method is described in more detail in Supporting Information and the fitting results are presented in *SI Appendix*, Fig. S8 and Table S5.

### UV-Visible and EPR Characterization of HgcAB Methylation.

To track the cobalamin states of HgcAB during Ti(III) citrate reduction and SAM methylation, the reaction was characterized in parallel by EPR and UV-visible spectroscopy. As-purified HgcAB (50 µM) was prepared in Buffer B. For UV-vis, samples were analyzed in a 1 mm path length cuvette. The Co(II) state of HgcAB was generated by incubating 50 µM HgcAB with 2 mM DTT for 120 min. To generate Cob(I)-HgcAB, 300 µM Ti(III) citrate was added to 50 µM HgcAB in Buffer B and incubated for 150 min at room temperature and monitored by UV-visible spectroscopy to verify the reduction. Then, to generate Me-Cob(III)-HgcAB, 300 µM Ti(III) citrate was added to 50 µM HgcAB in Buffer A for 150 min at room temperature, followed by addition of 200 µM SAM (final) in the dark.

### LC–MS Analyses of SAM and SAH, HgcAB, and MeHg.

The HgcAB-catalyzed conversion of SAM to SAH was also monitored by LC–MS. An anaerobic sample of 50 µM HgcAB in Buffer B was reduced to the Cob(I) state by reaction with 300 µM Ti(III) citrate. Then, 150 µM SAM was added in the dark, and the reaction was quenched after 5 min by addition of Quench Solution (methanol and formic acid) to precipitate the protein, the sample was centrifuged, and the supernatant was injected onto an Agilent LC–MS to separate and quantify SAM and SAH in group c of Fig. 2*E*. Meanwhile, two different control references were prepared similarly: one lacking SAM in group a of Fig. 2*E* and another with 200 µM SAM added after quenching in group b of Fig. 2*E*. A photolysis group d was also prepared to verify the synthesis of the Me-Cob(III)-HgcAB in which an anaerobic sample of 50 µM HgcAB in Buffer A was reduced to the Cob(I)-HgcAB state by 300 µM Ti(III) citrate. Then, 200 µM SAM was added, and the Eppendorf tube was exposed to a fluorescent lamp bulb (20 W/1,200 lm). After 30 min, an additional 200 µM Ti(III) citrate and 200 µM of SAM were included and the reaction was quenched after 30 min of exposure by adding Quench Solution. The protein was precipitated, and the sample was centrifuged in group d of Fig. 2*E*. All experiments were performed in triplicate and examined by LC–MS. The peaks assigned to SAM and SAH were integrated, the ratio of the integrations of SAM/(all peaks) and of SAH/(all peaks) were calculated for each sample and presented as ppm (parts per million) in Fig. 2*E*. Each lane represented the average value of each group of samples with error bars representing the SD.

### XAS Spectroscopy.

Purified HgcAB samples were loaded into XAS cuvettes sealed with 25 μm Kapton tape, flash frozen, and stored under LN2 until analysis at the Stanford Synchrotron Radiation Lightsource on beamline 9-3. During data collection, samples were maintained at 10 K using an Oxford Instruments CF 1208 liquid helium cryostat. Data were collected at the Co K-edges, and data were collected simultaneously on a Co foil standard for energy calibration. Spectra were collected at multiple spots per sample to minimize beam-induced damage, although no beam damage was observed across multiple scans on the same sample spot. Details of the XAS data analysis are provided in *SI Appendix* section.

## Supplementary Material

Appendix 01 (PDF)

## Data Availability

All study data are included in the article and/or *SI Appendix*.
