# Supplementary material for: S-adenosyl-L-methionine is the unexpected methyl donor for the methylation of mercury by the membrane-associated HgcAB complex
Source: Proc Natl Acad Sci U S A. 2024 Nov 15;121(47):e2408086121. doi: 10.1073/pnas.2408086121 (PMC11588087; doi:10.1073/pnas.2408086121)
Supplement: Supplementary file 1 — Appendix 01 (PDF) [file pnas.2408086121.sapp.pdf]

PNAS

Supporting Information for

S-adenosyl-L-methionine is the unexpected methyl donor for the methylation of mercury by the membrane-associated HgcAB complex

Kaiyuan Zheng[a,1], Katherine W. Rush[a,2], Swapneeta S. Date[b,3], Alexander Johs[b], Jerry M. Parks[c], Angela S. Fleischhacker[a], Macon J. Abernathy[d], Sarangi, Ritimukta[d], and Stephen W. Ragsdale[a*]

[a] Department of Biological Chemistry, University of Michigan Medical School, Ann Arbor, MI 48109-0606

[b] Environmental Sciences Division, Oak Ridge National Laboratory, Oak Ridge, TN 37831-6038

[c] Biosciences Division, Oak Ridge National Laboratory, Oak Ridge, TN 37831-6309

[d] Department of Structural Molecular Biology, Stanford Synchrotron Radiation Lightsource, SLAC National Accelerator Laboratory, Menlo Park, CA 94025

[1] Present Address: Department of Structural Molecular Biology, University of Virginia School of Medicine, Charlottesville, VA, 22908

[2] Present Address: Department of Chemistry and Biochemistry, Auburn University, Auburn, AL 36849-5312

[3] Present Address: Center for Technology Transfer and Commercialization, Vanderbilt University, Nashville, Tennessee, 37212-2838

Corresponding author name: Stephen W. Ragsdale
Email: sragsdal@umich.edu

This PDF file includes:

Full Experimental Details for the Materials and Methods Section
Figures S1 to S9
Tables S1 to S5

Cloning, expression and purification of HgcAB

To overcome the low native protein levels, we cloned the *hgcA* and *hgcB* genes from *P. mercurii* in tandem into *Escherichia coli (E. coli)* pETDuet-1 expression vector (Novagen, Madison, WI). The *hgcA* coding region was amplified by PCR from a custom synthesized, *E. coli* codon optimized *hgcA* vector (1). Then, it was cloned into the BamHI and HindIII restriction sites within MSC-1 of pETDuet-1 (Novagen, Madison, WI) for expression of HgcA with an N-terminal His_6-tag. Then, the coding region for a maltose-binding protein (MBP)-HgcB fusion protein was amplified from a previously described plasmid (1) and cloned into the NdeI and XhoI restriction sites within MSC-2 of the pETDuet-1 vector that already had *hgcA* in MSC-1. Finally, the MBP coding region was deleted using the Q5 site-directed mutagenesis kit (New England BioLabs, Ipswich, MA). Sequences were verified by Sanger sequencing (University of Michigan DNA Sequencing Core).

When expressed independently of HgcB, HgcA was unstable and insoluble, lacked cobalamin, and had very low expression levels. Similarly, HgcB expressed without HgcA was insoluble. However, expression of HgcA and HgcB in the tandem cloning sites of the pET duet vector produced a soluble HgcAB complex.

To ensure that the two [4Fe-4S] clusters were incorporated into HgcB and the cobalamin center into HgcA, we co-transformed the pRKisc vector for [4Fe-4S] cluster synthesis (2–4) and the pBtu vector for cobalamin uptake (5) with the HgcAB-containing pETDuet-hgcA-hgcB or pETDuet-His-hgcA-hgcB vector into both *E. coli* BL21(DE3) and C41(DE3) strains (6). In cell lysate assays for mercury methylation, constructs cloned into BL21(DE3) exhibited mercury methylation activity, while those cloned into C41(DE3) did not (not shown). Thus, we used the BL21(DE3) strain as our expression platform. After performing parallel experiments in BL21(DE3) under different conditions, we observed that the N-terminal His-tag does not significantly influence the HgcAB mercury methylation activity (compare rows 3 & 5 in Fig. 1C).

Anaerobic expression of HgcAB in M9-ETA media (components listed in Table S1) provided the highest activity (Fig. S1) (Table S3). Thus, we selected this growth condition to express the N-terminal His-tagged HgcA with a non-tagged HgcB for further purification and characterization. We used a 10 L fermenter (BIOFLO 110 Fementor/Bioreactor, New Brunswick Scientific, Edison Township, NJ) for HgcAB expression. The M9-ETA media was prepared and autoclaved in the fermenter; then ethanolamine, cobalamin, ampicillin (100mg/L), spectinomycin (50 mg/L), and tetracycline (10 mg/L) were added after the fermenter had cooled to approximately 22 °C (Table S1). Meanwhile, the BL21(DE3) strain containing HgcAB was precultured in 75 mL of M9-ETA media with ampicillin (100 mg/L) spectinomycin (50 mg/L) and tetracycline (10 mg/L) at 37℃, 225 rpm for 16 h in the incubation shaker (Innova® 44, New Brunswick Scientific, Edison Township, NJ). At an OD_{600} of 1.5, the preculture was inoculated into the 10L of M9-ETA media in the fermenter and grown initially under aerobic conditions at 37°C and 225 rpm until the OD_{600} reached 0.35, when 20 g of L-arabinose was added. When the OD_{600} reached 0.90, the media was sparged with high purity nitrogen gas for 1 h, and 3.6 g sodium dithionite (25 mL), 3.6 g of cysteine (25 mL), 0.5 g ampicillin (5 mL), 0.24 g isopropyl β-D-thiogalactoside (IPTG) (5 mL) and 2.0 g of ammonium iron sulfate (10 mL) were added via syringes for an additional 20 h at 37°C and 225 rpm. Finally, the cell pellets were centrifuged anaerobically at 5000×g for 15 min, and the cell mass was transferred into an anaerobic chamber and the cell mass determined.

Use of an N-terminal His_6-tag on HgcA enabled purification of the HgcAB complex using Ni-NTA agarose chromatography (Qiagen Ni-NTA agarose, Qiagen, Germantown, MD) (7). Because HgcA is a transmembrane protein, detergents were used to maintain solubility of the HgcAB complex. Our optimized purification protocol included 0.3% Sarkosyl and 0.6% Triton X-100, which enabled elution of homogeneous HgcAB from the Ni-NTA agarose.

All purification steps were performed in an anaerobic chamber (Vacuum Atmospheres). The cell pellets obtained from fermentation were resuspended in anaerobic Buffer A (50 mM K_2HPO_4, 100 mM NaCl, 10% glycerol, 1mM TCEP, pH 7.4) containing protease inhibitor (Roche Diagnostics GmbH, Mannheim, Germany), and 10 mM imidazole (10 mL buffer/g cell pellets). Next, the resuspended cell pellets were sonicated (MISONIX sonicators, Newtown, CT) (50 amplitude, 5 s on, 10 s off, 5 min process time followed by 10 min rest time per session, 4 sessions in total), the cell lysates anaerobically centrifuged at 26,500×g for 30 min, and the supernatants were collected for further anaerobic centrifugation at 89,400×g for 30 min. The supernatants were discarded and the precipitants containing the membrane debris was weighed and resuspended (0.13 g/mL) in

Buffer A with 1% Sarkosyl, 2% Triton X-100, 10 mM imidazole for 1 h at room temperature. Then, 2.3 volumes of Buffer A was added for another sonication (50 amplitude, 5 s on, 10 s off, 5 min process time followed by 10 min rest time per session, 2 sessions in total). The sonicated mixture was anaerobically centrifugated at 89,400×g for 30 min and the supernatant was applied to a Ni-NTA agarose column pre-equilibrated with Buffer A containing 0.3% Sarkosyl, 0.6% Triton X-100. Then, the column was washed with same buffer, then Buffer A (lacking Sarkosyl and Triton X-100), and Buffer A containing 500 mM imidazole; finally, the pure HgcAB complex was eluted via elution buffer (Buffer A containing 0.15% sarkosyl, 0.15% Triton X-100, 250 mM imidazole). The resulting HgcAB complex was typically >95% pure, containing the expected 37 kDa HgcA and the 11 kDa HgcB Coomassie-stained protein bands by SDS-PAGE (Fig. S2).

The HgcAB complex was buffer exchanged using an Amicon centrifugal filter unit (Amicon® Ultra - 0.5 mL 30 kDa, Amicon, Miami, FL) and dialyzed overnight against Buffer B (20 mM HEPES, 150 mM NaCl, 1 mM TCEP, 10% glycerol, pH 7.4). To prepare HgcAB samples for XAS characterization, an additional EDTA dialysis step was included to remove Ni contamination that interferences XAS signal. After buffer exchanged using an Amicon centrifugal filter unit, HgcAB samples were dialyzed overnight against Buffer C (20 mM HEPES, 150 mM NaCl, 1 mM TCEP, 1 mM EDTA, 0.1% Sarkosyl, 10% glycerol, pH 7.4). Then the HgcAB samples were dialyzed overnight against Buffer B (20 mM HEPES, 150 mM NaCl, 1 mM TCEP, 10% glycerol, pH 7.4). The purified HgcAB complex stoichiometrically bound cobalamin (8, 9) (Fig. S3A and S3B) and two [4Fe-4S] clusters. MS/MS identification of SDS-PAGE gel slices validating the identity of the 37 and 11 kDa bands (Fig. S2), and purified protein provided ~90% coverage for both HgcA and HgcB (Fig. S3C).

P. mercurii culture conditions and preparation of cell lysates

WT *P. mercurii* (formerly *Desulfovibrio desulfuricans* ND132) or Δ*hgcAB* cultures (ND132 cells lacking the *hgcA* and *hgcB* genes) were prepared as described previously (10) and were grown in modified minimal organic basal medium with yeast extract (MOY) (11) containing 40 mM fumarate, 40 mM pyruvate, and 1.2 mM thioglycolate and 1 mM cysteine·HCl as reducing agents for approximately 3 days (OD_{600} = 0.3) at 32°C under anaerobic conditions. Cell pellets were harvested at 4 °C by centrifugation for 20 min at 7,500×g in centrifuge bottles with sealing closures. Then, the cell pellets were washed 2 times with deoxygenated phosphate buffered saline (PBS), pH 7.0, and resuspended in cold lysis buffer containing Pierce™ EDTA-free protease inhibitor (Thermo Fisher Scientific, Waltham, MA), Benzonase® Nuclease (MilliporeSigma, St. Louis, MO), and 2 mM dithiothreitol (DTT) in PBS, pH 7.0 and disrupted on ice by sonication under strictly anaerobic conditions inside a glove box under an N_2 atmosphere (O_2 ≤ 1 ppm) with minimal exposure to ambient light. Unlysed cells were removed by centrifugation at 30,000×g for 1h at 4°C. Total protein concentration of cell lysates was determined by the Bradford method with bovine serum albumin (BSA) (Millipore Sigma, St. Louis, MO) as standard and the lysates were stored in aliquots at -80 °C in amber glass vials with PTFE-lined silicone caps.

HgcAB analytical assays

Protein was determined using the BCA assay (Pierce™ BCA Protein Assay Kit, Thermo Fisher Scientific, Waltham, MA). The dialyzed protein sample was diluted 50-fold in Buffer B (20 mM HEPES, 150 mM NaCl, 1 mM TCEP, 10% glycerol, pH 7.4). Then the concentration of the diluted protein solution was assayed according to the User Guide of the Pierce™ BCA Protein Assay Kit and based on a standard curve generated by bovine serum albumin.

The cobalamin content of HgcAB was determined as the dicyano-Co(III)balamin complex by treatment of the protein solution with KCN at 95℃ (12). Representative spectra of as-purified HgcAB and derivatized dicyano-cob(III)alamin can be found in Figure S3B and S3A, respectively. The protein was diluted approximately 10-fold with Buffer B to reach a final dicyano-Co(III)balamin concentration between 2-20 μM (best linearized range). Then, 5 μL of potassium cyanide (1 M KCN, 0.25 mM NaOH) was added into 50 μL of the diluted protein sample. Then the solution was heated to 95°C for 20 min in Eppendorf tubes using heat blocks for 20 min, centrifuged at 17,000 x g for 15 min and the cobalamin concentration was determined by UV-visible spectroscopy using extinction coefficients of 26,200, 7,400, and 8,600 $M^{-1}cm^{-1}$ at 367, 540, and 580 nm (8, 9). All

HgcAB concentrations reported below and throughout the paper are similarly based on their cyano-Co(III)balamin content.

The HgcAB [4Fe-4S] cluster occupancy was determined by electron paramagnetic resonance (EPR). The protein in Buffer B was diluted to 50 μM and the [4Fe-4S] clusters were reduced by addition of 100mM sodium dithionite (1mM, final) for 2 h at room temperature. Then, 150 μL of sodium dithionite-reduced HgcAB (49.5 μM) was transferred into a quartz EPR tube and characterized by EPR spectroscopy under the following conditions: temperature, 10K; microwave power, 10 mW; microwave frequency, 9.38 GHz; receiver gain, 44,800; modulation amplitude, 5.0 G; modulation frequency, 100 kHz. The EPR spectrum (Fig. S4) indicated a coupled $[4Fe\text{-}4S]^{+}$ system, consistent with a classic 8 iron ferredoxin (13, 14). The spin concentration was measured by double integration referenced to a 1 mM copper perchloride standard according to Eq. 3, where DI is the double integral over the entire derivative signal; gp is the average *g*; MA is the modulation amplitude; power is the power in W; gain is the spectrometer gain setting (15). The Cu(II) spectrum was performed using the following conditions: temperature, 10 K; microwave power, 10 mW; microwave frequency, 9.38 GHz; receiver gain, 44,800; modulation amplitude, 1.0 G; modulation frequency, 100 kHz. All EPR spectra were collected through Bruker EMX WinEPR Acquisition Software, and double integrated through the Bruker WinEPR Post Processing Software.

(Eq. 3)

$$[4Fe-4S](\boldsymbol{\mu}M) = Cu(\boldsymbol{\mu}M) \cdot \frac{DI([4Fe-4S])}{DI(Cu)} \frac{gp(Cu)}{gp([4Fe-4S])} \frac{MA(Cu)}{MA([4Fe-4S])} \sqrt{\frac{power(Cu)}{power([4Fe-4S])}} \frac{gain(Cu)}{gain([4Fe-4S])}$$

Mercury methylation cell lysate assays

These experiments were designed to produce <50 pmol of MeHg and performed in special facilities at ORNL to ensure safety. Cell lysates of *E. coli* strains containing the overexpressed HgcAB complex were prepared from frozen washed cell pellets after resuspension in lysis buffer and sonication under anaerobic conditions, as described above. After removal of unlysed cells by centrifugation and determination of the total protein concentration. Hg methylation assays were conducted in an anaerobic glove box under a pure N_2 atmosphere ($[O_2]$ < 1 ppm) in the dark. Samples were added to 4 mL amber glass vials and the total protein concentration was adjusted with anoxic PBS buffer to 1.5 mg/mL. For experiments in which cell lysates were mixed, aliquots of the *E. coli* lysates were added to a *P. mercurii* Δ*hgcAB* cell lysate, to a protein concentration of 0.1 mg/mL unless specified otherwise.

To study the effect of metabolites on Hg methylation in ND132 cell lysates, freshly prepared stocks (1.5 mM) of 5-methyltetrahydrofolate (Me-THF), S-adenosyl methionine (SAM), acetyl-CoA (acetyl coenzyme A sodium salt), L-glycine, L-methionine, or L-serine (MilliporeSigma, St. Louis, MO) were added to Hg methylation assays at a final concentration of 30 μM and in a final volume of 1 mL, or at increasing concentrations in establishing the K_m for SAM.

In all mercury methylation assays, samples were equilibrated for 5 min before adding an aliquot of a freshly prepared $HgCl_2$ stock solution (1.5 μM) to a final concentration of 30 nM and incubated at 32 °C for 2 hours. Then, the reactions were stopped by adding 0.5% (v/v) trace-metal grade H_2SO_4 (Thermo Fisher Scientific, Waltham, MA), and the samples were moved immediately to a -20 °C freezer and stored until MeHg analysis by inductively coupled plasma mass spectrometry (ICP-MS) as described below.

Replicate samples were taken out of the glove box chamber, and aliquots (0.5 mL) were analyzed for MeHg using a modified EPA Method 1630 via distillation and ethylation (16, 17). Modifications to the EPA methods included the use of isotope dilution with enriched stable isotopes to determine total Hg and methylmercury concentrations, and detection of Hg by ICP-MS to separate the various Hg isotopes (18).

Single turnover assays of HgcAB methylation

To track the HgcAB-catalyzed cobalamin methylation reaction, we performed the enzymatic assay in Buffer B (20 mM HEPES, 150 mM NaCl, 1 mM TCEP, 10% glycerol, pH 7.4). A titanium(III) citrate (Ti(III) citrate) solution was prepared by adding 100 μL of 15% Ti(III) chloride into a premixed buffer containing 1125 μL of 0.5 M sodium citrate and 400 μL of 1 M Tris buffer,

pH 8.0 for 10 min at room temperature. An aliquot was diluted into Buffer B to 66 mM Ti(III) citrate for cobalamin reduction.

Methylation assays were performed using 10 μM HgcAB in Buffer B. Then 100 μM Ti(III) citrate (final) was added to reduce the as-purified Cob(III)- to Cob(I)-HgcAB and appearance of the 390 nm Co(I) peak was monitored. Next, 50 μM methyl donor was added and disappearance of the 390 nm Co(I) peak and appearance of a broad 479 nm peak were monitored.

The non-enzymatic methylation of cobalamin was performed similarly using 10 μM reduced hydroxocobalamin in Buffer B. Each step of the reaction was tracked by UV-vis spectrometry. To thoroughly reduce the 10 μM of hydroxocobalamin, 400 μM Ti(III) citrate was added for a 20 min reaction, and reduction was monitored by appearance of the 390 nm Co(I) peak in the UV-vis spectrum. Meanwhile, the excess Ti(III) citrate significantly increased the baseline absorbance (Fig. S5A). Then 50 μM SAM was added and the 390 nm Co(I) peak slowly disappeared in 20 min, concomitant with the appearance of a broad 520 nm peak (Fig. S5B).

HgcAB stopped-flow kinetic assays

To characterize the kinetics for the HgcAB methylation reaction, stopped flow UV-vis experiments were performed at 21 °C within an anaerobic glove box (Vacuum Atmospheres) under a pure N_2 atmosphere (O_2 < 1 ppm) on an Applied Photophysics (Leatherhead, England). spectrophotometer (SX18MV with the Pro-Data upgrade) equipped with monochromator and photodiode array (PDA) detector. In one syringe (Reagent A), 10 μM HgcAB in Buffer A was reduced by addition of 100 μM Ti(III) citrate (final). The other syringe (Reagent B) contained SAM (100 μM, 200 μM, 400 μM, 800 μM, 1200 μM, 1600 μM, or 2000 μM) in the same buffer. Reagents A and B were mixed via a 1:1 ratio in single-mixing mode and the experiments were performed in triplicate. UV-vis spectra were collected from 300 nm to 700 nm for analysis and fitting. An example is shown in Fig. 2D, in which data were acquired at 50 μM SAM after mixing and spectra from different time points of the reaction were superimposed.

The stopped flow data were acquired using the Pro-data SX software. Each set of reactions was monitored for 600 s and 1000 spectra were collected in a logarithmic time interval. Analysis was performed using Pro-data viewer software and data at both 390 nm and 479 nm were fit to a double exponential equation (Fig. S7) to yield both k_{fast} and k_{slow} (Table S4). The fitting of k_{fast} obtained from both 390 nm and 479 nm versus SAM concentration was performed using Graphpad Prism 9 software (Fig. S8D). The kinetic data from 0.05 to 5 s at 390 nm and 479 nm were fit to a two-step reversible model via KinTek Explorer 11.1.0 software, to obtain the rate constants k_1, k_2, k_3, and k_4.

The KinTek Explorer 11.1.0 software was used for kinetic data fitting. Kinetic data at 390 nm and 479 nm, ranging from 0.05s to 600s, were imported into KinTek Explorer with a time offset of 0.05s. A two-step reversible mechanism model was established as E + S = ES = EP + B, where E represents Cob(I)-HgcAB, S represents SAM, ES represents SAM·HgcAB, EP represents Me-Cob(III)-HgcAB, and B represents SAH. Two experiments were set up in the Experiment Editor: one for the 390 nm kinetic traces and another for the 479 nm kinetic traces. In both experiment setups, E was set to 5, S was set to 50, 100, 200, 400, 600, 800, and 1000, and Time was set to 5 in the Mixing Step 1 [t=0] section. In the Observables section, S1_c in the 390 nm experiment was set as a1*(E+ES)+c1, and S1_c in the 479 nm experiment was set as b2*EP+c2. The Conc. Series Offset (add/subtract) option was selected for data normalization. In the Data Repository, the aFit-Estimated Sigma (per experiment) option was selected for Sigma Options per Experiment. Kinetic data at 390 nm and 479 nm were fitted individually and globally to acquire kinetic parameters k_1, k_2, k_3, and k_4 along with their standard errors. The fitting results are presented in Fig. S8 and Table S5.

UV-visible and EPR characterization of HgcAB methylation

To track the cobalamin states of HgcAB during Ti(III) citrate reduction and SAM methylation, the reaction was characterized in parallel by EPR and UV-visible spectroscopy. As-purified HgcAB (50 μM) was prepared in Buffer B. For UV-vis, samples were analyzed in a 1 mm path length cuvette. EPR experiments were performed in quartz tubes using the following conditions: sample volume, 200 μL; temperature, 60K and 10K; microwave power, 10 mW; microwave frequency, 9.38 GHz; receiver gain, 44,800; modulation amplitude, 5.0 G or 1.0 G;

modulation frequency, 100 kHz. EPR spectra were recorded on a Bruker EMX spectrometer (Bruker Biospin Corp., Billerica, MA) equipped with an Oxford ITC4 temperature controller, a Hewlett-Packard model 5340 frequency counter, and a Bruker gaussmeter. The Co(II) state of HgcAB was generated by incubating 50 μM HgcAB with 2 mM dithiothreitol (DTT) for 120 min. To generate Cob(I)-HgcAB, 300 μM Ti(III) citrate was added to 50 μM HgcAB in Buffer B and incubated for 150 min at room temperature and monitored by UV-visible spectroscopy to verify the reduction. Then, to generate Me-Cob(III)-HgcAB, 300 μM Ti(III) citrate was added to 50 μM HgcAB in Buffer A for 150 min at room temperature, followed by addition of 200 μM SAM (final) in the dark.

LC-MS analyses of SAM and SAH, HgcAB, and MeHg

The HgcAB-catalyzed conversion of SAM to SAH was also monitored by LC-MS. An anaerobic sample of 50 μM HgcAB in Buffer B was reduced to the Cob(I) state by reaction with 300 μM Ti(III) citrate. Then, 150 μM SAM was added in the dark and the reaction was quenched after 5 min by addition of Quench Solution (methanol and formic acid) to precipitate the protein, and the sample was centrifuged at 13,300×g for 10 min. Then the supernatant was injected onto an Agilent LC-MS to separate and quantify SAM and SAH in group c of Fig. 2E. Meanwhile, 2 different control references were prepared similarly: one lacking SAM in group a of Fig. 2E and another with 200 μM SAM added after quenching in group b of Fig. 2E. A photolysis group d was also prepared to verify the synthesis of the Me-Cob(III)-HgcAB in which an anaerobic sample of 50 μM HgcAB in Buffer A was reduced to the Cob(I)-HgcAB state by 300 μM Ti(III) citrate. Then, 200 μM SAM was added and the Eppendorf tube was exposed to a fluorescent lamp bulb (20W/1200lm). After 30 min, an additional 200 μM Ti(III) citrate and 200 μM of SAM was included and the reaction was quenched after 30 min of exposure by adding Quench Solution. The protein was precipitated, and the sample was centrifuged at 13300×g for 10 min in group d of Fig. 2E. All experiments were performed in triplicate and examined by LC-MS. The peaks assigned to SAM and SAH were integrated, the ratio of the integrations of SAM/(all peaks) and of SAH/(all peaks) were calculated for each sample and presented as ppm (parts per million) in Fig. 2E. Each lane represented the average value of each group of samples with error bars representing the standard deviation.

LC-MS analysis was performed on an Agilent 6520 Accurate-Mass Q-TOF LC/MS system (Agilent, Santa Clara, CA). The Agilent MassHunter Workstation MS software was used for data acquisition and analysis. Reversed-phase HPLC was performed on an Agilent 1260 Infinity Quaternary LC System (Agilent, Santa Clara, CA) equipped with an Agilent Poroshell 120 4.6×100 mm 2.7 μm EC-C18 column (Agilent, Santa Clara, CA). The chromatographic method used for characterizing SAM and SAH consisted of the following gradient of water with 15% methanol (solvent A) and water (solvent B), each containing 0.1% formic acid: 0% B for 9 min, 0-50% B over 1 min, 50% B for 4 min, 50-0% B over 1 min, 0% B for 15 min at a flow rate of 0.1 mL/min. The electrospray ionization (ESI) mass detector was configured to positive ion mode with scanning between 50-3200 *m/z*. SAM and SAH were identified and searched in MS data via *m/z* peaks at 399.14 and 385.13.

Verification of the HgcAB sequence was obtained by LC/MS/MS. The HgcAB sample was cleaned up using an S-Trap kit (Protifi, Fairport, NY) according to the manufacturer's protocol. Then, the sample was reduced with DTT; alkylated with iodoacetamide and incubated at 37℃ overnight in the presence of trypsin. The resulting peptides were extracted and analyzed by nano LC/MS/MS (Orbitrap Velos, Thermo Scientific, Waltham, MA). The digested sample was loaded and gradient eluted over an Agilent 10 cm 75 μm ID C18 column at 400 nL/min for a 30min gradient. The mass spectrometer was operated in data-dependent mode; the seven most abundant ions were selected for MS/MS. MS/MS data were searched using a local copy of Mascot (www.matrixscience.com). The data were processed and visualized via Scaffold viewer software.

Methylmercury was measured with an automated MERX purge and trap system (Brooks Rand Instruments, Seattle, WA) followed by detection on an inductively coupled plasma mass spectrometer (ICP-MS, ELAN DRC-e, PerkinElmer Inc., Shelton, CT). Replicate samples were taken out of the glove chamber, and aliquots (0.5 mL) were analyzed for MeHg using a modified EPA Method 1630 via distillation and ethylation (16, 17). Modifications to the EPA methods included the use of isotope dilution with enriched stable isotopes to determine total Hg and methylmercury concentrations, and detection of Hg by ICP-MS to separate the various Hg isotopes (19).

X-ray Absorption Spectroscopy (XAS)

Purified HgcAB samples were loaded into solution XAS cuvettes sealed with 25 µm Kapton tape, flash frozen and stored under LN2 until analysis at the beamline. X-ray absorption spectra were collected at the Stanford Synchrotron Radiation Lightsource on beamline 9-3, a 2T 20-pole wiggler side station. Fluorescence data were collected using a monolithic 100 element Ge detector (Canberra). Energy selection was provided by a double Si(220) crystal monochromater oriented to ϕ = 0, and harmonic rejection was provided by a spherically bent, Rh-coated mirror. Unwanted signal from photon scattering was reduced using a Mn filter and Soller slits. During data collection, samples were maintained at a temperature of 10 K using an Oxford Instruments CF 1208 liquid helium cryostat. Data were collected at the Co K-edges, and data were collected simultaneously on a Co foil standard for energy calibration. Spectra were collected at multiple spots per sample to minimize beam-induced damage, although no beam damage was observed across multiple scans on the same sample spot.

Due to the dilute nature of the samples, and a large background signal from ice diffraction observed in some of the channels, the fluorescence data were analysed channel-by-channel (each channel representing a single detector element of the 100-element Ge detector) using Larch (20) to identify and exclude the compromised channels. Channels that were found to be satisfactory were then summed for each scan and imported to Athena (21) for calibration and normalization. Spectra of the reference foil were calibrated to 7709 eV, and the energy shift was then applied to the corresponding sample spectrum. The normalized data were then transferred to Pyspline (22) where the EXAFS spectra were extracted by setting the value of E_0 to 7730 and fitting a 4-region spline to the data with polynomial orders 2, 3, 3, and 3, and in the case of the EXAFS exceeding 12 $Å^{-1}$, a 5th 3rd order polynomial region was added. Least-squares fitting of the EXAFS data was done in Artemis (21). Feff8 (20) was used to generate theoretical EXAFS phase and amplitude parameters of scattering paths within a 5 Å radius of the Co absorber using the DFT optimized structures as input. Four parameters were evaluated for each backscattering path in course of fitting: the interatomic distance R, the interatomic disorder σ^2, the shift in the assigned value of E_0, and the amplitude reduction factor S_0^2. Of these four parameters, R and σ^2 were allowed to float for each interaction, while the shift in E_0 was allowed to float but fixed to a common value among all paths in a given fit. S_0^2 was fixed at 0.9. Voigt modelling of the pre-edge feature was done using Larch v9.45 from 7704 eV to 7715 eV, with the peak range defined as 7707 eV to 7713.5 eV. A baseline composed of a linear and Lorentzian component (the default in this version of Larch) was fit prior to fitting the Voigt function. The FWHM was kept at a value of 1.9 for all spectra. XAS DFT computational details are provided in the SI.

XAS computational details

All DFT calculations were performed using Orca 5.0.3 (23). Cys-on structures utilized the structural model of HgcAB developed by Cooper et al. (24), and structures without Cys ligation utilized the crystal structure of methyl cob(III)alamin published by Randaccio et al (25) as input structures. These structures were edited using Avogadro v1.2.0 (26) as needed to create the models with aquo trans-axial ligand and cbi species. Gradient-corrected (GGA), spin-unrestricted Kohn-Sham orbitals were calculated using the BP86 functional (27, 28), which has been shown to do well at reproducing the geometry of methylcobalamin (29). The def2-TZVP basis set of the Karlsruhe group was used on all atoms, except Co, on which the Ahlrichs doubly polarized triple-ζ basis set was used (30), and the auxiliary basis set def2/J was used in the Coulomb fitting (31). The default atom-pairwise dispersion correction with the Becke-Johnson damping scheme (D3BJ) was also implemented (32, 33). Solvation effects were accounted for through the water SMD solvation model (34). Tight SCF convergence criteria were enforced, and a default grid scheme defgrid2 was used for all atoms while a radial integration grid of 7 set for Co.

Time-dependent DFT calculations on the geometry optimized structures were performed at the same level of theory. Thirty transitions were calculated for each TD-DFT spectrum, which were Gaussian broadened using a FWHM of 1.7 eV to account for instrument resolution and core-hole lifetime effects. The calculated spectra were linearly scaled such that the maximum height of the methylcob(III)alamin matched that of the experimental data. The energies were likewise linearly shifted by 196.4 eV.

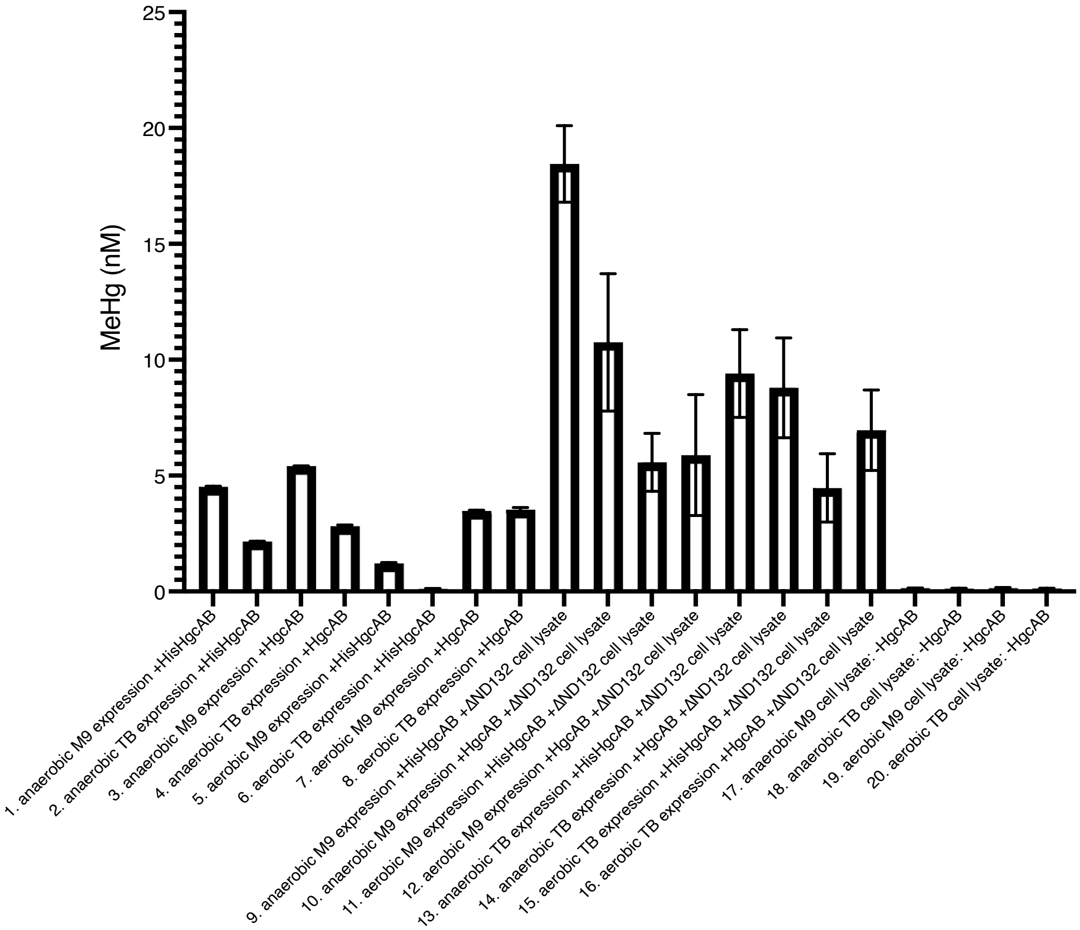

Fig. S1. Enzymatic mercury methylation assay for cell lysate from *E. coli* and *Pseudodesulfovibrio mercurii* ND132 Δ*hgcAB*. Mercury methylation assays results for *E. coli* and *P. mercurii* ND132 Δ*hgcAB* cell lysates under aerobic/anaerobic conditions, TB/M9 media, and using His-tagged/non-tagged HgcA. Error bars represent standard deviations. N=3 for each bar. Details of assays conditions are described in Materials and Methods, Supporting Information, and Table S3.

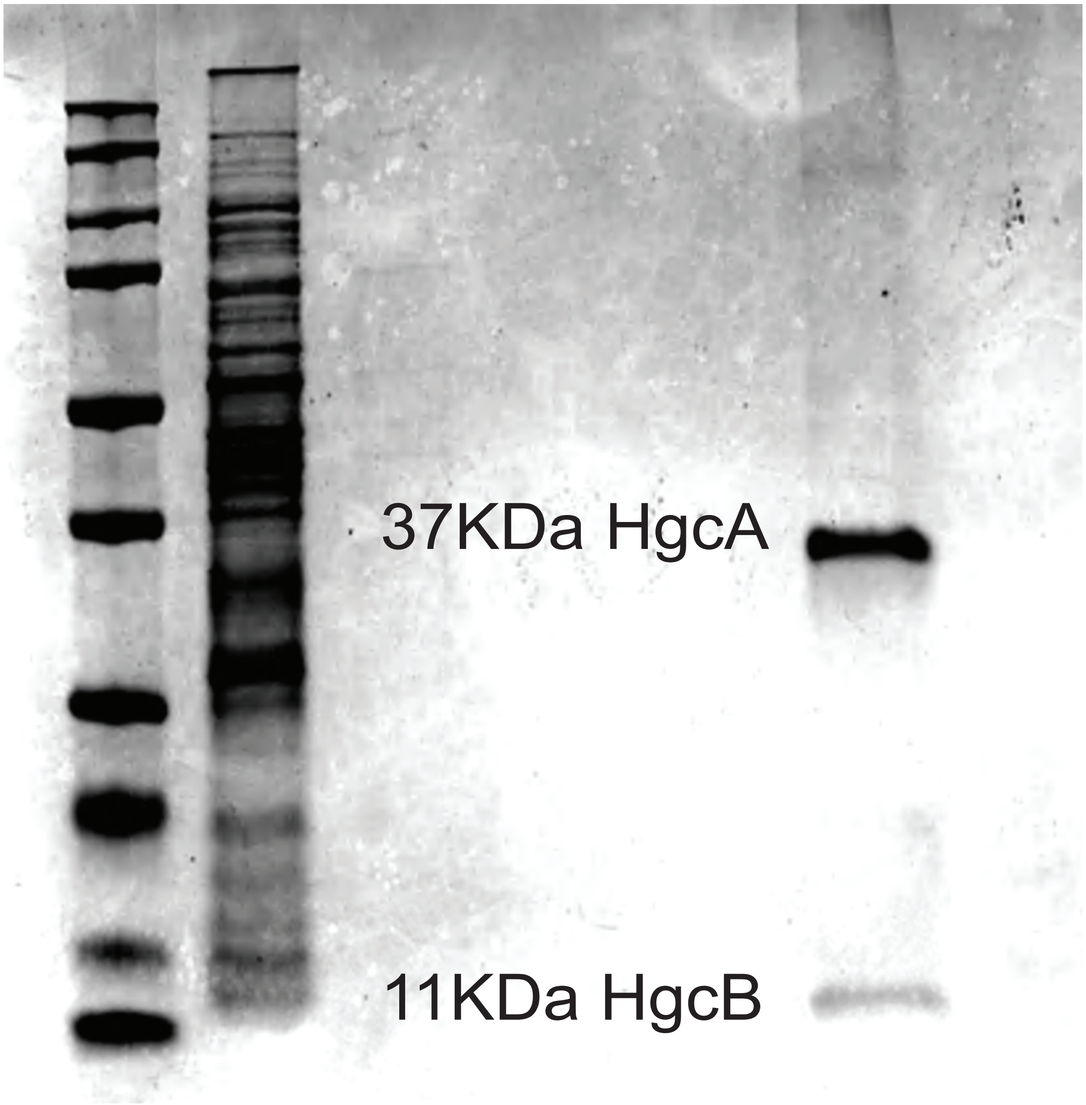

Fig. S2. SDS-PAGE gel of purified HgcAB. N-terminal His-tagged HgcA and HgcB are purified as a complex via Ni-NTA column. The 37 kDa band represents the HgcA and the 11 kDa band represents the HgcB (lane 3). Lane 1 is the ladder marker, and lane 2 is the flow through of the Ni-NTA column.

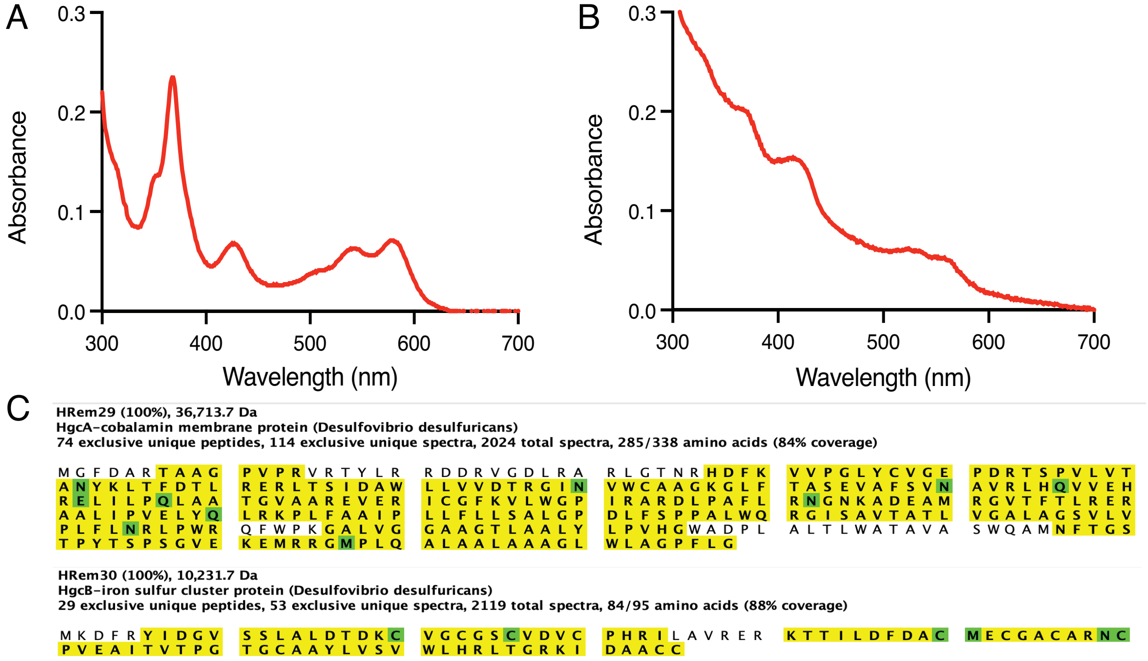

Fig. S3. UV-visible spectra and MS/MS coverage of purified HgcAB. (A) UV-vis spectrum of dicyano-Co(III)balamin assays results of purified HgcAB. (B) UV-vis spectrum of as-purified Co(III)-HgcAB. (C) MS/MS coverage of purified HgcAB. Experimental details can be found in Materials and Methods and Supporting Information.

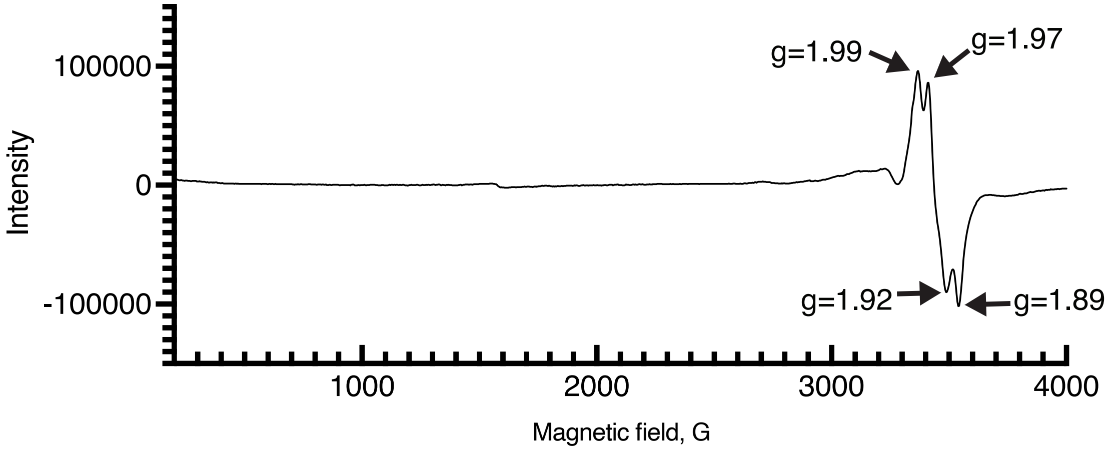

Fig. S4, EPR spectrum of [4Fe-4S]$^+$ HgcAB EPR spectrum of 50 µM of [4Fe-4S]$^+$ HgcAB. 50 µM HgcAB was reduced by 1 mM sodium dithionite. Four main peaks in EPR signal are labeled, g=1.99, 1.97, 1.92, and 1.89. The EPR spectrum was acquired at 10 K, see Materials and Methods section for additional details.

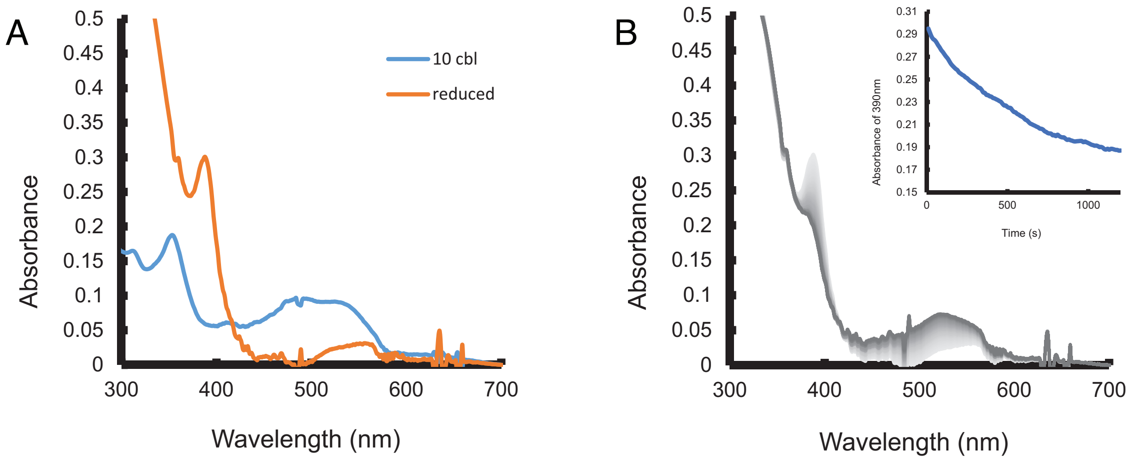

Fig. S5. UV-visible spectra and kinetic data of hand-mixing Cob(III)alamin non-enzymatic methylation test using SAM. (A) UV-vis spectra of 10 µM of Cob(III)balamin and then reduced to Cob(I)alamin by adding 400 µM titanium(III) citrate. (B) UV-vis kinetic spectra of Cob(I)alamin from Fig. S5A after reaction with 50 µM SAM and monitored for 20 min at a 10 s interval. The top right corner figure shows the absorbance decrease at 390 nm peak in Fig. S5B. Experimental details can be found in the Materials and Methods.

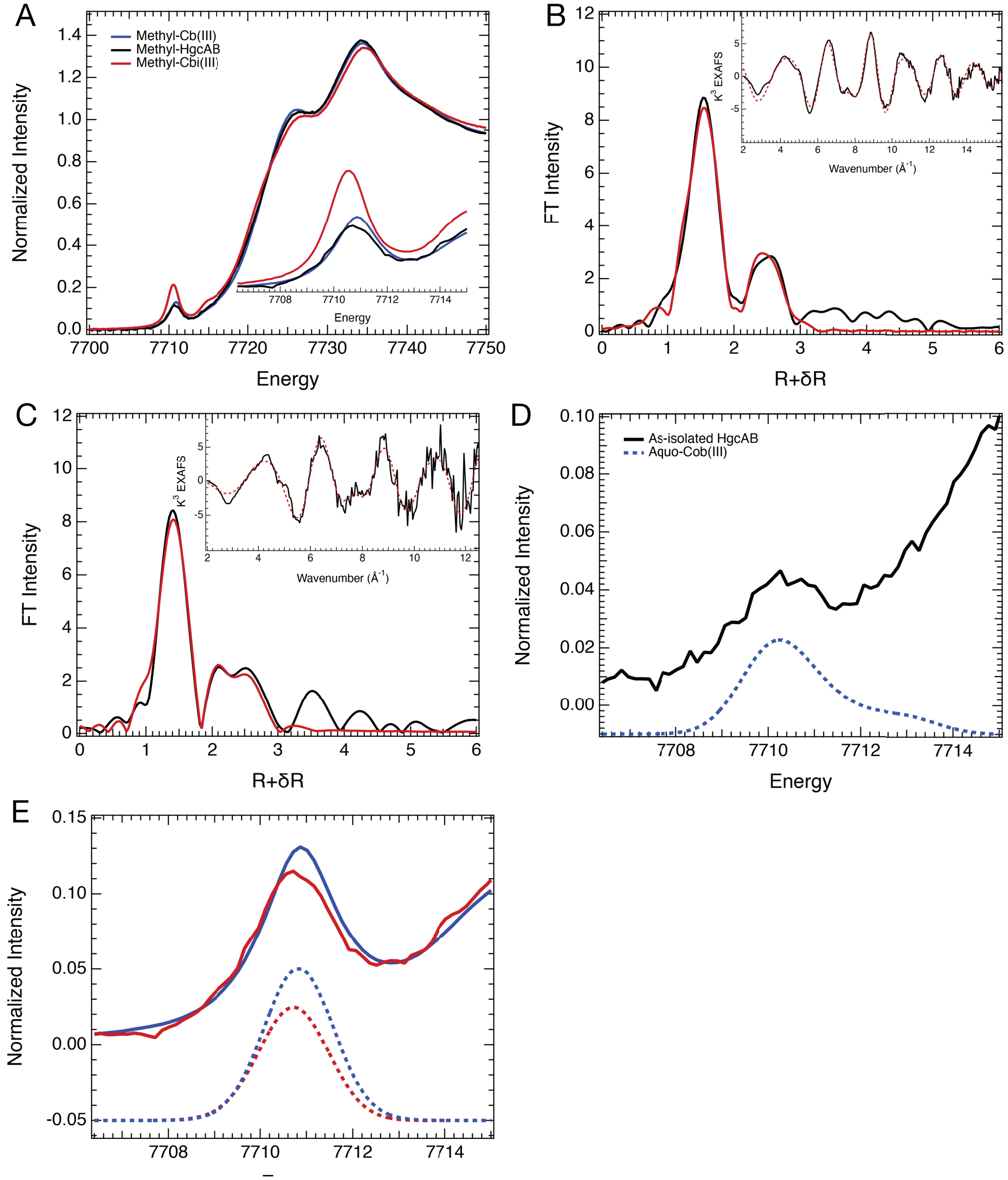

Figure S6. Co K-edge XAS. (A) Comparison of the Co K-edge XAS data for Me-HgcAB (black) with methyl-cob(III)alamin (blue) and methyl-cob(III)inamide (red). (B) Non-phase-shift corrected Fourier transform and (inset) corresponding EXAFS for methyl-Cob(III)alamin. Data (black), fit (red). (C) Non-phase-shift corrected Fourier transform and (inset) corresponding EXAFS for as-isolated HgcAB. Data (black), fit (red). (D) Comparison of the pre-edge feature of the As-isolated HgcAB with TD-DFT simulations of the expected pre-edge features of aquo-cob(III)alamin. The simulated spectrum has been scaled and shifted as described in the methods section. (E) The Co K-pre-edge XAS data for Me-HgcAB (red) and methylcob(III)alamin (blue) (solid lines) and their corresponding TD-DFT simulations (dashed lines). The simulated spectra have been scaled and shifted as described in the Materials and Methods section and computational details can be found in the Supporting Information.

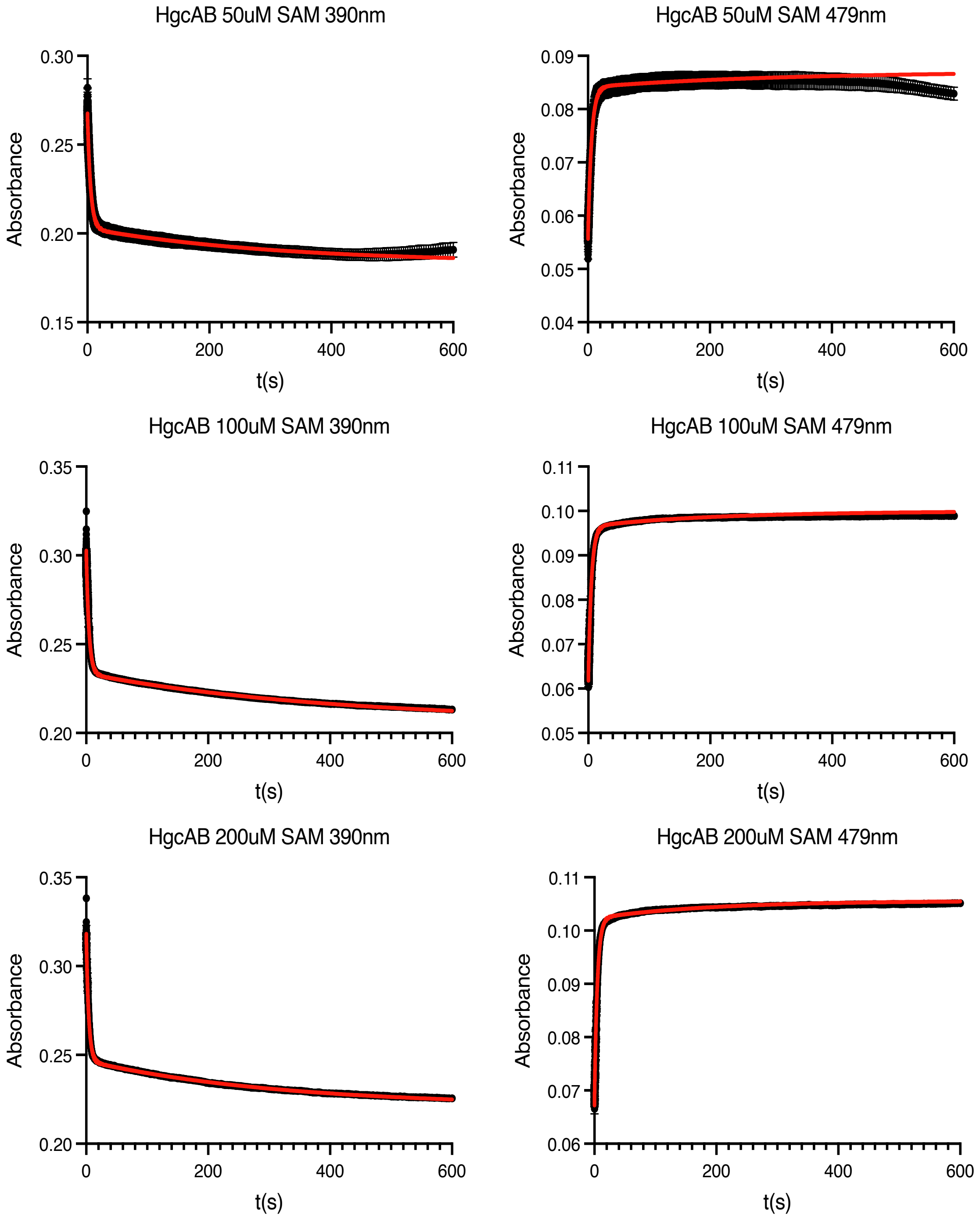
HgcAB 50uM SAM 390nm
Absorbance
0.30
0.25
0.20
0.15
0
200
400
600
t(s)
HgcAB 50uM SAM 479nm
Absorbance
0.09
0.08
0.07
0.06
0.05
0.04
0
200
400
600
t(s)
HgcAB 100uM SAM 390nm
Absorbance
0.35
0.30
0.25
0.20
0
200
400
600
t(s)
HgcAB 100uM SAM 479nm
Absorbance
0.11
0.10
0.09
0.08
0.07
0.06
0.05
0
200
400
600
t(s)
HgcAB 200uM SAM 390nm
Absorbance
0.35
0.30
0.25
0.20
0
200
400
600
t(s)
HgcAB 200uM SAM 479nm
Absorbance
0.11
0.10
0.09
0.08
0.07
0.06
0
200
400
600
t(s)

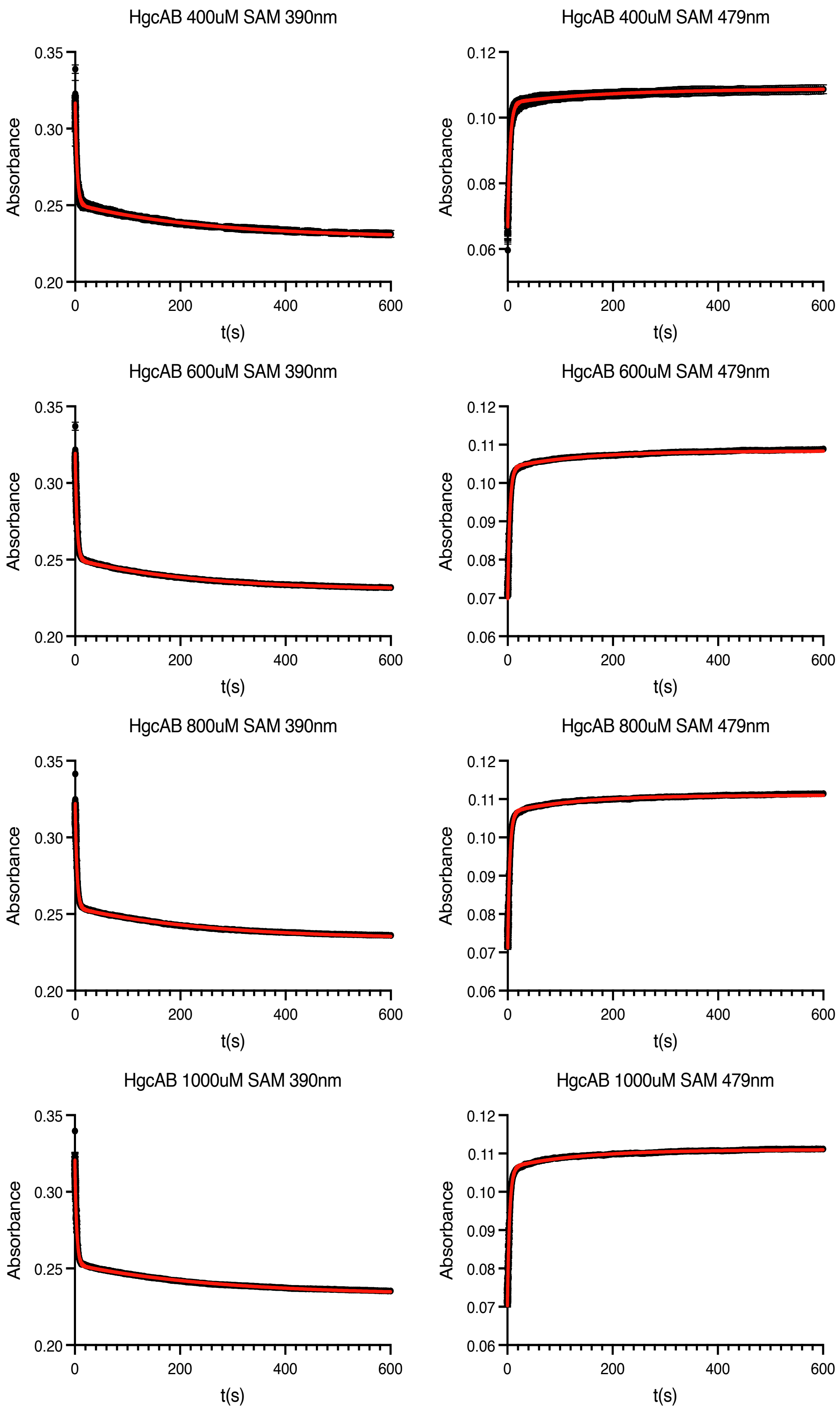
HgcAB 400uM SAM 390nm
HgcAB 400uM SAM 479nm
HgcAB 600uM SAM 390nm
HgcAB 600uM SAM 479nm
HgcAB 800uM SAM 390nm
HgcAB 800uM SAM 479nm
HgcAB 1000uM SAM 390nm
HgcAB 1000uM SAM 479nm
Absorbance
t(s)

Fig. S7. Stopped flow studies of SAM methylation of HgcAB plotted at 390 nm and 479 nm. UV-vis kinetics tracking of Cob(I)-HgcAB SAM methylation reaction. 5 μM of Cob(I)-HgcAB was reacted with varying concentrations of SAM and UV-vis spectra were acquired. Experimental data are represented as black lines and fitted data represented by red lines. Experimental details can be found in Materials and Methods.

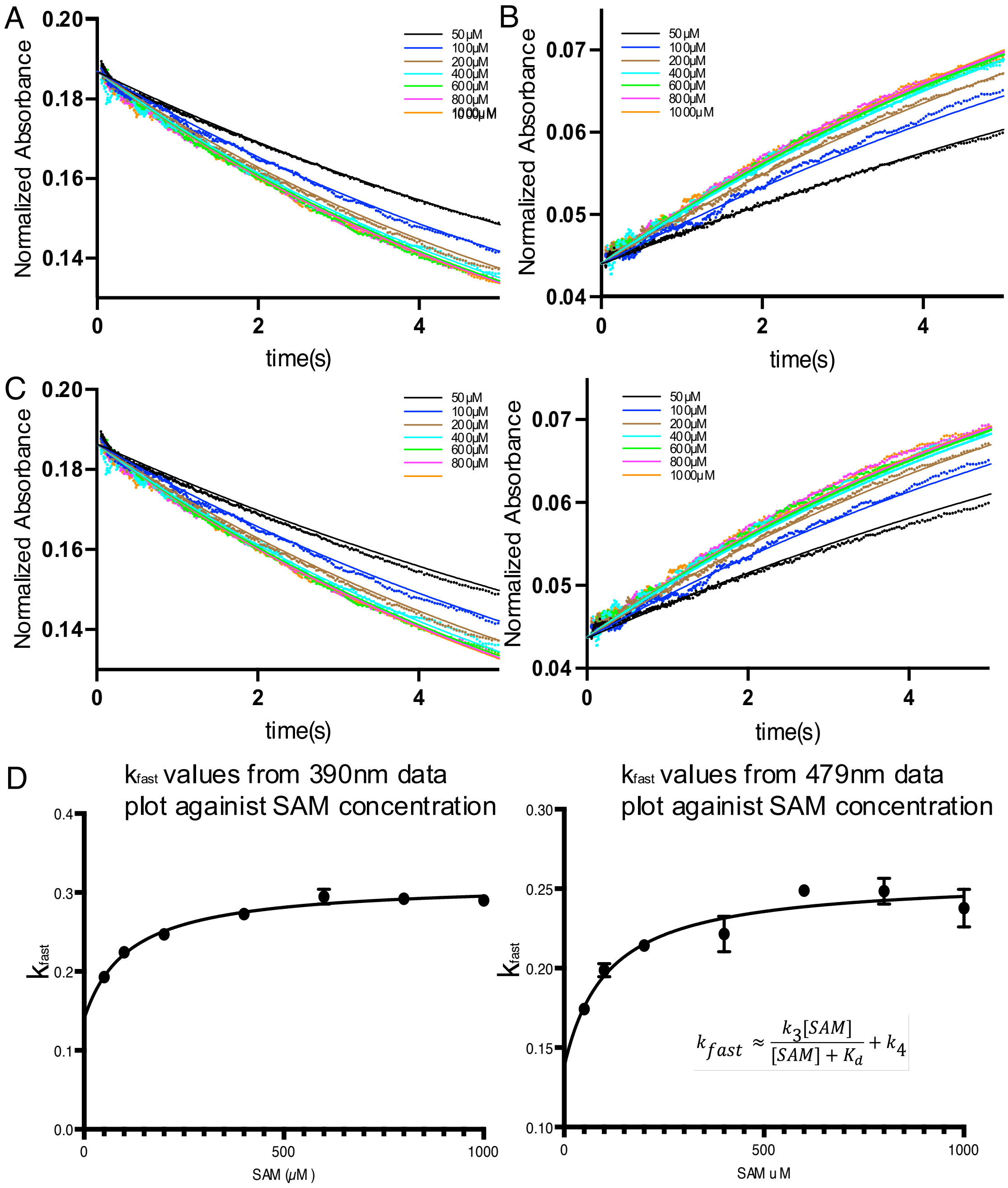

Fig. S8. Kinetics fitting of SAM methylation of Cob(I)-HgcAB. (A) The normalized 390 nm kinetics data, acquired using stopped-flow spectroscopy, were fitted to a two-step reversible model using KinTek Explorer. The resulting rate constants k_1, k_2, k_3, and k_4 were 0.55 ± 0.04 L· μmol^{-1}·s^{-1}, 16 ± 1 s^{-1}, 0.160 ± 0.003 s^{-1}, and 0.002 ± 0.001 μmol^{-1}·s^{-1}. (B) The normalized 479 nm kinetics data, also acquired using stopped-flow spectroscopy, were fitted to a two-step reversible model using KinTek Explorer. The resulting rate constants k_1, k_2, k_3, and k_4 were 0.40 ± 0.09 L· μmol^{-1}·s^{-1}, 16 ± 4 s^{-1}, 0.13 ± 0.02 s^{-1}, and 0.002 ± 0.01 μmol^{-1}·s^{-1}. (C) Both the normalized 390 nm and 479 nm kinetics data, acquired using stopped-flow spectroscopy, were globally fitted to a two-step reversible model using KinTek Explorer. The left panel shows the original data and fitting results for the 390 nm data, while the right panel shows the corresponding information for the 479

nm data. The resulting rate constants k_1, k_2, k_3, and k_4 are 0.48 ± 0.09 L·μmol^{-1}·s^{-1}, 16 ± 3 s^{-1}, 0.15 ± 0.02 s^{-1}, and 0.002 ± 0.009 μmol^{-1}·s^{-1}. Different concentrations of SAM are represented by different colors, with the dispersed spots indicating the normalized data points and the solid lines representing the fitting results. Detailed fitting results are reported in Table S5. (D) The k_{fast} values, obtained from double exponential equation fitting (Eq. 2) of the original 390 nm (left panel) and 479 nm (right panel) kinetics data, are plotted against increasing SAM concentration. The data were fitted using the equation shown in the right panel, which represents a form obtained after several approximations. The spots indicate the averaged k_{fast} values for different SAM concentrations, with each error bar representing the standard deviation for each k_{fast} value. The solid curve represents the fitting curve. Detailed fitting results are reported in Table S4.

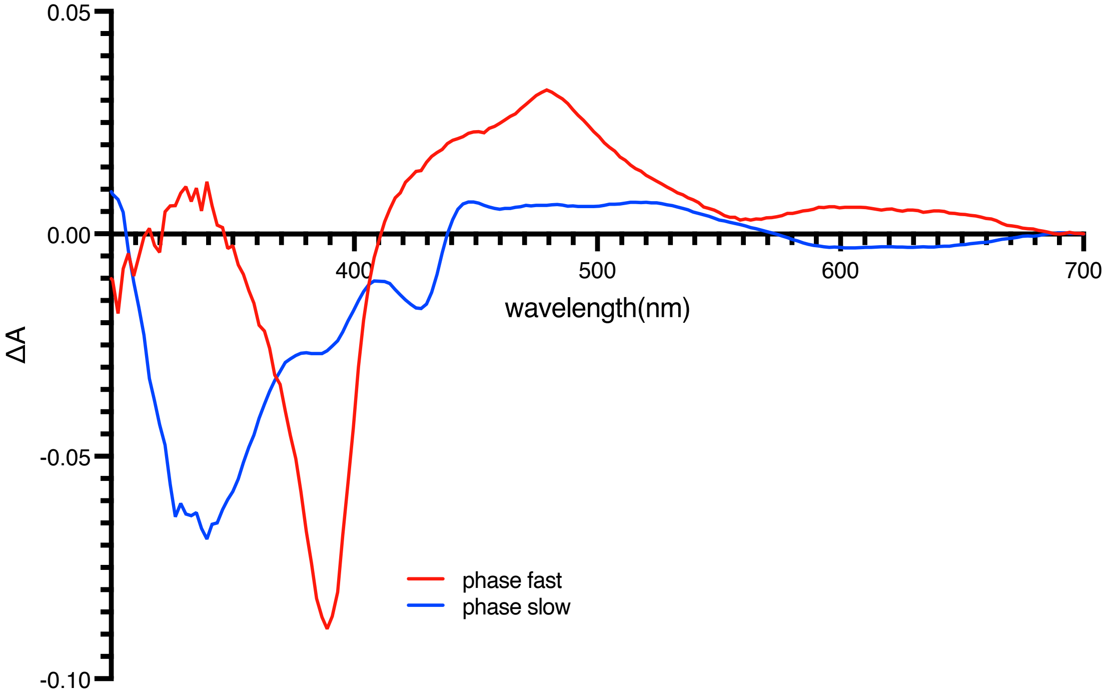

Fig. S9. UV-vis differential spectra associated with the fast and slow phases of the reactions after SAM addition to Cob(I)-HgcAB. 50 μM SAM added to 5 μM Cob(I)-HgcAB. The red trace represents UV-vis spectrum acquired after 10 s of reaction subtracted from the initial UV-vis spectrum before reaction. The blue trace represents the UV-vis spectrum acquired after 600 s of reaction subtracted from that acquired after 10 s.

Table S1. M9 media recipe for HgcAB expression.

10X M9 Salts (1 L)	
Na_2HPO_4-$12H_2O$*	171.5 g
KH_2PO_4	30 g
NaCl	5 g
ddH2O	to 1 L

**1L 10,000X Micronutrient Mixture**	
Ingredient	mg/L
$(NH_4)_2Mo_7O_{24}$	37.1
$CoCl_2 \cdot 6H_2O$	71.4
H_3BO_3	247
$CuSO_4 \cdot 5H_2O$	25
$MnCl_2 \cdot 4H_2O$	198
$ZnSO_4 \cdot 7H_2O$	28.8

**500 mL Cobalamin Supplement†**	
Ingredient	Quantity
10,000X Micronutrient Mixture	4 mL
$MgSO_4 \cdot 7H_2O$	9.2 g
$CaCl_2 \cdot 2H_2O$	0.6 g
Thiamine	37 mg
Hydroxocobalamin	126 mg

**1M Ethanolamine†**	
Ingredient	Quantity
1M HCl	940 mL
Ethanolamine	60 mL
pH to 7.4 w/ HCl or NaOH	

**Amino Acid Supplement for 10L media**			
Ingredient	MW	[Media] (mM)	for 10L (g)
Ala	89.09	0.8	0.713
Arg	210.7	0.4	0.843
Asn	132.1	0.4	0.5285
Asp	133.1	0.4	0.5325
Cys	121.2	0.1	0.121
Glu	147.1	0.6	0.8825
Gln	146.2	0.6	0.8775
Gly	75.07	0.8	0.6005
His	209.6	0.2	0.4195
Ile	131.2	0.4	0.525
Leu	131.2	0.8	1.0495
Lys	182.7	0.4	0.731
Met	149.2	0.2	0.2985
Phe	165.2	0.4	0.661
Pro	115.1	0.4	0.4605
Ser	105.1	10	10.51
Thr	119.1	0.4	0.4765
Trp	204.2	0.1	0.2045
Tyr	181.2	0.2	0.3625
Val	117.2	0.6	0.7035

**(10L) M9-ETA media**				
M9 Salts 10x	1L		1M Ethanolamine	250ml
Cobalamin Supplement	135ml		Ampicilin	1g
Spectinomycin	0.5g		tetracyclin	0.1g

Table S2. Assays conditions of mercury methylation tests in Fig. 1*

Lane #	Component 1, [protein]	Component 2 (ND132 Δ*hgcAB* extract), 0.1 mg/mL	Component 3, concentration	N-terminal His-tag HgcA
1	-	+	-	-
2	*E. coli* cell lysate, 1.5 mg/mL	-	-	+
3	*E. coli* cell lysate, 1.5 mg/mL	-	MeTHF, 150 μM	-
4	*E. coli* cell lysate, 1.5 mg/mL	-	SAM, 150 μM	-
5	*E. coli* cell lysate, 1.5 mg/mL	-	MeTHF, 150 μM	+
6	*E. coli* cell lysate, 1.5 mg/mL	-	SAM, 150 μM	+
7	*E. coli* cell lysate, 1.5 mg/mL	+	MeTHF, 150 μM	-
8	*E. coli* cell lysate, 1.5 mg/mL	+	SAM, 150 μM	-
9	*E. coli* cell lysate, 1.5 mg/mL	+	MeTHF, 150 μM	+
10	*E. coli* cell lysate, 1.5 mg/mL	+	SAM, 150 μM	+
11	*E. coli* membrane debris 1.5 mg/mL	+	MeTHF, 150 μM	+
12	*E. coli* membrane debris, 1.5 mg/mL	+	SAM, 150 μM	+
13	purified HisHgcA HgcB, 0.1 mg/mL	+	MeTHF, 150 μM	+
14.	purified HisHgcA HgcB, 0.1 mg/mL	+	SAM, 150 μM	+
15	methylated HisHgcA HgcB, 0.1 mg/mL	-	-	+
16	*E. coli* cell lysate, 1.5 mg/mL	+	Sarkosyl, 0.3%	+
17	*E. coli* cell lysate, 1.5 mg/mL	+	-	+
18	*E. coli* cell lysate, 1.5 mg/mL	+	SAM, 150μM	+

*Experimental details can be found in Materials and Methods and Supporting Information.

Table S3. Assay conditions of mercury methylation tests in Fig. S1*

Lane	1.5 mg/mL *E. coli* cell lysate Component 1	0.1 mg/mL ND132 Δ*hgcAB* cell lysate Component 2	pETDuet-hgcA-hgcB vector	His-tag HgcA	media	Anaerobic (+) Aerobic (-)
1	+	-	+	+	M9	+
2	+	-	+	+	TB	+
3	+	-	+	-	M9	+
4	+	-	+	-	TB	+
5	+	-	+	+	M9	-
6	+	-	+	+	TB	-
7	+	-	+	-	M9	-
8	+	-	+	-	TB	-
9	+	+	+	+	M9	+
10	+	+	+	-	M9	+
11	+	+	+	+	M9	-
12	+	+	+	-	M9	-
13	+	+	+	+	TB	+
14.	+	+	+	-	TB	+
15	+	+	+	+	TB	-
16	+	+	+	-	TB	-
17	+	-	-	-	M9	+
18	+	-	-	-	TB	+
19	+	-	-	-	M9	-
20	+	-	-	-	TB	-

* All assays are performed with extracts of *E. coli* cells harboring pBtu and pRKisc plasmids as described in Materials and methods.

Table S4. Stopped flow kinetics fitted parameters of HgcAB SAM methylation reaction at 390 nm and 479 nm

**390 nm data**, $k_{fast}(obs)$					
[SAM], μM	kobs (1)	kobs (2)	kobs (3)	$k_{fast}(obs)$, avg.	$k_{fast}(obs)$, *SD
50	0.197	0.190	0.191	0.193	0.004
100	0.223	0.220	0.230	0.224	0.005
200	0.248	0.246	0.248	0.247	0.001
400	0.271	0.271	0.276	0.273	0.003
600	0.304	0.295	0.285	0.295	0.009
800	0.292	0.289	0.299	0.292	0.007
1000	0.289	0.296	0.285	0.290	0.005
**390 nm data**, $k_{slow}(obs)$					
[SAM], μM	kobs (1)	kobs (2)	kobs (3)	$k_{slow}(obs)$, avg.	$k_{slow}(obs)$, *SD
50	0.0033	0.0029	0.0034	0.0032	0.0002
100	0.0025	0.0024	0.0024	0.0025	0.0000
200	0.0029	0.0033	0.0032	0.0031	0.0002
400	0.0038	0.0036	0.0047	0.0040	0.0006
600	0.0044	0.0044	0.0044	0.0044	0.0000
800	0.0040	0.0035	0.0042	0.0039	0.0003
1000	0.0035	0.0036	0.0038	0.0037	0.0002
**479 nm data**, $k_{fast}(obs)$					
SAM], μM	kobs (1)	kobs (2)	kobs (3)	$k_{fast}(obs)$, avg.	$k_{fast}(obs)$, *SD
50	0.175	0.175	0.173	0.174	0.001
100	0.201	0.201	0.194	0.199	0.004
200	0.215	0.212	0.216	0.214	0.002
400	0.233	0.221	0.211	0.221	0.011
600	0.247	0.249	0.251	0.249	0.002
800	0.246	0.257	0.242	0.248	0.008
1000	0.249	0.239	0.226	0.238	0.012
**479 nm data**, $k_{slow}(obs)$					
SAM], μM	kobs (1)	kobs (2)	kobs (3)	$K_{slow}(obs)$, avg.	$k_{slow}(obs)$, *SD
50	0.0027	0.0034	0.0033	0.0032	0.0004
100	0.0079	0.0030	0.0057	0.0055	0.0025
200	0.0061	0.0047	0.0051	0.0053	0.0007
400	0.0074	0.0056	0.0027	0.0053	0.0023
600	0.0058	0.0085	0.0086	0.0076	0.0016
800	0.0081	0.0102	0.0058	0.0081	0.0022
1000	0.0093	0.0095	0.0049	0.0079	0.0026

Experimental details can be found in Materials and Methods. *SD, standard deviation.

Table S5. KinTek Explorer fitted kinetics parameters for HgcAB SAM methylation reaction at 390 nm, or 479 nm, or 390 nm and 479 nm globally

**KinTek Explorer simulation fitting results for 390 nm kinetics data**		
Sigma w.r.t fit		0.00080318
Chi square		2397.78
Chi square/DoF		1.10446
P-value		0
Chi square threshold		0.994222
**Parameters**	**Best Fit**	**Standard Error**
k_1	0.552731	0.0447973
k_2	16.1453	1.30509
k_3	0.160225	0.00367605
k_4	0.00172449	0.00142123
**KinTek Explorer simulation fitting results for 479 nm kinetics data**		
Sigma w.r.t fit		0.000464179
Chi square		2407.89
Chi square/DoF		1.10912
P-value		0
Chi square threshold		0.994222
**Parameters**	**Best Fit**	**Standard Error**
k_1	0.398372	0.0877063
k_2	16.3645	3.71986
k_3	0.130287	0.0208543
k_4	0.00154702	0.0128233
**KinTek Explorer simulation global fitting results for both 390 nm and 479 nm kinetics data**		
Sigma w.r.t fit		0.00072479
Chi square		5976.54
Chi square/DoF		1.37518
P-value		0
Chi square threshold		0.996441
**Parameters**	**Best Fit**	**Standard Error**
k_1	0.479929	0.0885433
k_2	16.2151	3.15462
k_3	0.147258	0.0153351
k_4	0.00159535	0.00850743

References

1. K. W. Rush, “Expression and Characterization of HgcA and HgcB, Two Proteins Involved in Methylmercury Biosynthesis,” University of Michigan.

2. M. Nakamura, K. Saeki, Y. Takahashi, Hyperproduction of Recombinant Ferredoxins in Escherichia coli by Coexpression of the ORF1-ORF2-iscS-iscU-iscA-hscB-hscA-fdx-ORF3 Gene Cluster. *J. Biochem. (Tokyo)* **126**, 10–18 (1999).

3. D. C. Johnson, D. R. Dean, A. D. Smith, M. K. Johnson, STRUCTURE, FUNCTION, AND FORMATION OF BIOLOGICAL IRON-SULFUR CLUSTERS. *Annu. Rev. Biochem.* **74**, 247–281 (2005).

4. C.-W. Lin, J. W. McCabe, D. H. Russell, D. P. Barondeau, Molecular Mechanism of ISC Iron–Sulfur Cluster Biogenesis Revealed by High-Resolution Native Mass Spectrometry. *J. Am. Chem. Soc.* **142**, 6018–6029 (2020).

5. N. D. Lanz, *et al.*, Enhanced Solubilization of Class B Radical *S* -Adenosylmethionine Methylases by Improved Cobalamin Uptake in *Escherichia coli*. *Biochemistry* **57**, 1475–1490 (2018).

6. C. T. Chung, S. L. Niemela, R. H. Miller, One-step preparation of competent Escherichia coli: transformation and storage of bacterial cells in the same solution. *Proc. Natl. Acad. Sci.* **86**, 2172–2175 (1989).

7. A. Spriestersbach, J. Kubicek, F. Schäfer, H. Block, B. Maertens, “Purification of His-Tagged Proteins” in *Methods in Enzymology*, (Elsevier, 2015), pp. 1–15.

8. R. A. Firth, *et al.*, The chemistry of vitamin B12. Part IX. Evidence for five-co-ordinate cobalt(III) complexes. *J. Chem. Soc. Inorg. Phys. Theor.* 2419 (1968).

9. C. Giannotti, *B12* (Wiley-Interscience New York, 1982).

10. S. S. Date, *et al.*, Kinetics of Enzymatic Mercury Methylation at Nanomolar Concentrations Catalyzed by HgcAB. *Appl. Environ. Microbiol.* **85**, e00438-19 (2019).

11. S. D. Smith, *et al.*, Site-Directed Mutagenesis of HgcA and HgcB Reveals Amino Acid Residues Important for Mercury Methylation. *Appl. Environ. Microbiol.* **81**, 3205–3217 (2015).

12. J. Maillard, *et al.*, Characterization of the Corrinoid Iron-Sulfur ProteinTetrachloroethene Reductive Dehalogenase of *Dehalobacterrestrictus*. *Appl. Environ. Microbiol.* **69**, 4628–4638 (2003).

13. R. Mathews, S. Charlton, R. H. Sands, G. Palmer, On the nature of the spin coupling between the iron-sulfur clusters in the eight-iron ferredoxins. *J. Biol. Chem.* **249**, 4326–4328 (1974).

14. J. R. Baur, M. C. Graves, B. A. Feinberg, S. W. Ragsdale, Characterization of the recombinant Clostridium pasteurianum ferredoxin and comparison of its properties with those of the native protein. *BioFactors Oxf. Engl.* **2**, 197–203 (1990).

15. J. A. Fee, "[20] Transition metal electron paramagnetic resonance related to proteins" in *Methods in Enzymology*, (Elsevier, 1978), pp. 512–528.

16. J. M. Parks, *et al.*, The Genetic Basis for Bacterial Mercury Methylation. *Science* **339**, 1332–1335 (2013).

17. X. Yin, *et al.*, Synergistic Effects of a Chalkophore, Methanobactin, on Microbial Methylation of Mercury. *Appl. Environ. Microbiol.* **86**, e00122-20 (2020).

18. Y. Cai, O. C. Braids, Eds., *Biogeochemistry of Environmentally Important Trace Elements* (American Chemical Society, 2002).

19. X. Lu, *et al.*, Anaerobic Mercury Methylation and Demethylation by *Geobacter bemidjiensis* Bem. *Environ. Sci. Technol.* **50**, 4366–4373 (2016).

20. A. L. Ankudinov, B. Ravel, J. J. Rehr, S. D. Conradson, Real-space multiple-scattering calculation and interpretation of x-ray-absorption near-edge structure. *Phys. Rev. B* **58**, 7565–7576 (1998).

21. B. Ravel, M. Newville, *ATHENA* , *ARTEMIS* , *HEPHAESTUS* : data analysis for X-ray absorption spectroscopy using *IFEFFIT*. *J. Synchrotron Radiat.* **12**, 537–541 (2005).

22. A. Tenderholt, B. Hedman, K. O. Hodgson, PySpline: A Modern, Cross-Platform Program for the Processing of Raw Averaged XAS Edge and EXAFS Data in *AIP Conference Proceedings*, (AIP, 2007), pp. 105–107.

23. F. Neese, F. Wennmohs, U. Becker, C. Riplinger, The ORCA quantum chemistry program package. *J. Chem. Phys.* **152**, 224108 (2020).

24. C. J. Cooper, *et al.*, Structure determination of the HgcAB complex using metagenome sequence data: insights into microbial mercury methylation. *Commun. Biol.* **3**, 320 (2020).

25. L. Randaccio, *et al.*, Similarities and Differences between Cobalamins and Cobaloximes. Accurate Structural Determination of Methylcobalamin and of LiCl- and KCl-Containing Cyanocobalamins by Synchrotron Radiation. *Inorg. Chem.* **39**, 3403–3413 (2000).

26. M. D. Hanwell, *et al.*, Avogadro: an advanced semantic chemical editor, visualization, and analysis platform. *J. Cheminformatics* **4**, 17 (2012).

27. A. D. Becke, Density-functional exchange-energy approximation with correct asymptotic behavior. *Phys. Rev. A* **38**, 3098–3100 (1988).

28. J. P. Perdew, Density-functional approximation for the correlation energy of the inhomogeneous electron gas. *Phys. Rev. B* **33**, 8822–8824 (1986).

29. H. Hirao, Which DFT Functional Performs Well in the Calculation of Methylcobalamin? Comparison of the B3LYP and BP86 Functionals and Evaluation of the Impact of Empirical Dispersion Correction. *J. Phys. Chem. A* **115**, 9308–9313 (2011).

30. F. Weigend, R. Ahlrichs, Balanced basis sets of split valence, triple zeta valence and quadruple zeta valence quality for H to Rn: Design and assessment of accuracy. *Phys. Chem. Chem. Phys.* **7**, 3297–3305 (2005).

31. F. Weigend, Accurate Coulomb-fitting basis sets for H to Rn. *Phys. Chem. Chem. Phys.* **8**, 1057 (2006).

32. S. Grimme, J. Antony, S. Ehrlich, H. Krieg, A consistent and accurate *ab initio* parametrization of density functional dispersion correction (DFT-D) for the 94 elements H-Pu. *J. Chem. Phys.* **132**, 154104 (2010).

33. S. Grimme, S. Ehrlich, L. Goerigk, Effect of the damping function in dispersion corrected density functional theory. *J. Comput. Chem.* **32**, 1456–1465 (2011).

34. A. V. Marenich, C. J. Cramer, D. G. Truhlar, Universal Solvation Model Based on Solute Electron Density and on a Continuum Model of the Solvent Defined by the Bulk Dielectric Constant and Atomic Surface Tensions. *J. Phys. Chem. B* **113**, 6378–6396 (2009).
